# Integrating UHPLC-QE-MS and Bioinformatics with Experimental Validation Reveals MAPK/FOS-Mediated Podocyte Apoptosis as the Key Mechanism of *Alpiniae oxyphyllae* and *Saposhnikovia divaricata* in Treating Diabetic Kidney Disease

**DOI:** 10.3390/ph18101449

**Published:** 2025-09-27

**Authors:** Xian Wang, Lin Zhang, Rongxin Tang, Wenlong Zhang, Yiqiang Xie, Kai Li

**Affiliations:** School of Traditional Chinese Medicine, Hainan Medical University, Haikou 571199, China; bancheng@muhn.edu.cn (X.W.); zhanglynner@muhn.edu.cn (L.Z.); tangrongxin@muhn.edu.cn (R.T.); zhang_wenlong@muhn.edu.cn (W.Z.)

**Keywords:** diabetic kidney disease, *Alpiniae oxyphyllae-Saposhnikoviae divaricata*, podocyte apoptosis, MAPK/FOS signaling pathway

## Abstract

**Background:*** Alpiniae oxyphyllae-Saposhnikovia divaricata* (AS), a traditional Chinese dietary supplement, exhibits potential therapeutic effects against diabetic kidney disease (DKD), though its active compounds and mechanisms require elucidation. **Methods:** Animal experiments integrated with UHPLC-QE-MS, bioinformatics, and experimental validation were employed to investigate AS’s pharmacodynamic basis against DKD. **Results:** Thirty-nine compounds were identified in AS, including four key flavonoids (daidzein, kaempferol, tectoridin, baicalin). Bioinformatics screening revealed 516 potential AS targets from PubChem/TCMSP/ETCM databases. Analysis of the GEO dataset (GSE30529) identified 482 DKD-related differentially expressed genes (DEGs). Venny 2.1 analysis yielded 42 co-DEGs and 6 co-core DEGs. Functional enrichment (GO/KEGG/GSEA) demonstrated AS’s modulation of apoptosis and extracellular matrix (ECM) pathways via these DEGs. ROC profiling and renal single-cell sequencing highlighted FOS as a specific regulator of podocyte apoptosis in DKD. Molecular docking confirmed stable binding between the four flavonoids and FOS. Experimentally, AS significantly suppressed expression of ECM-related proteins (Col-IV, LN, IL-6, IL-17) and pro-apoptotic proteins (Bax, Caspase-3), while restoring anti-apoptotic Bcl-2 levels and inhibiting phosphorylation of MEK4, JNK1, c-Jun, and FOS in DKD mice. **Conclusion:** This study elucidates that AS alleviates DKD by inhibiting the MAPK/FOS pathway, thereby attenuating podocyte apoptosis and ECM accumulation. These findings establish a foundation for targeted AS therapy in DKD.

## 1. Introduction

Diabetes mellitus (DM) is a prevalent metabolic disorder exhibiting a rising global prevalence, posing a significant “global threat” [1]. In 2021, the number of individuals affected by DM worldwide reached 500 million (10.5% of the global population), and this figure is projected to rise to nearly 800 million (12.2%) by 2045 [2]. Approximately 30% (type 1) to 40% (type 2) of DM patients develop Diabetic Kidney Disease (DKD) within 10 to 20 years of disease onset [3]. DKD represents a common microvascular complication in DM patients and is a leading cause of end-stage renal disease (ESRD) [4]. The pathogenesis of DKD is complex, involving alterations in multiple metabolic and hemodynamic factors. Pathologically, DKD is closely associated with renal fibrosis, accumulation of extracellular matrix (ECM) components in renal cells, basement membrane thickening, and chronic inflammation [5]. Although evidence supporting the use of emerging therapies like SGLT2 inhibitors for DKD continues to grow, these agents are associated with side effects such as hyperkalemia and transient renal dysfunction [6]. Furthermore, existing research predominantly employs surrogate endpoints like urinary protein levels and target organ protection, often overlooking the subjective symptoms and quality of life issues experienced by DKD patients [7].

The long-term course of DKD requires that the intervention strategies should be both safe and sustainable, and dietary management is the core part of this. In January 2024, the American Diabetes Association (ADA) updated its standards for “Improving Care and Promoting Health in Populations,” providing recommendations for six key health behaviors [8]. Within these, nutritional therapy offers guidance for the rational clinical application and development of dietary supplements for patients with DKD [9]. The National Institutes of Health (NIH) recognizes the significant role of Traditional Chinese herbs in dietary supplements due to their richness in natural bioactive compounds [10]. In East Asia, *Alpiniae oxyphyllae* and *Saposhnikovia divaricata* are common raw materials for herbal tea beverages or dietary supplements [11,12]. For centuries, they have been widely used as dietary supplements in the health regulation of patients with chronic diseases. Based on the dietary characteristics of *Alpiniae oxyphyllae* and *Saposhnikovia divaricata* and the clinical experience of Traditional Chinese medicine, we have found that the combined use of *Alpiniae oxyphyllae-Saposhnikovia divaricata* (AS) can effectively be applied to the daily management of DKD patients [13]. Our prior research has demonstrated that the AS combination possesses potential benefits in ameliorating renal fibrosis and inflammation in DKD patients, a mechanism potentially linked to the modulation of inflammatory cytokines and the NF-κB signaling pathway [14]. In vitro experimental systems have further confirmed the synergistic advantage of this dual-herb supplement combination for DKD [15]. Separate studies revealed that the therapeutic efficacy of *Alpiniae oxyphyllae* in DKD mice models is associated with its ability to improve gut microbiota and renal pathology in DKD animal models [16]. Concurrently, *Saposhnikovia divaricata* has been explicitly reported to effectively lower uric acid levels and ameliorate renal impairment [17].

Nevertheless, as a natural herbal dietary supplement, the bioactive compounds of AS and its in-depth, well-defined mechanisms of action for treating DKD remain to be further elucidated. In recent years, apoptosis of intrinsic renal cells, recognized as an early pathological phenotype in DKD, has garnered increasing attention for its impact on renal ECM homeostasis, inflammatory responses, and fibrotic processes [18]. This study, taking the foundational efficacy of AS against DKD and its active compounds as the starting point, combined bioinformatics analysis with experimental validation, elucidates the mechanism by which AS regulates podocyte apoptosis and ECM metabolism in DKD mice via the mitogen-activated protein kinase/FOS (MAPK/FOS) signaling pathway.

## 2. Results

### 2.1. AS Ameliorates Renal Function and Attenuates Podocyte and Tubular Injury in db/db Mice

**Figure 1:**
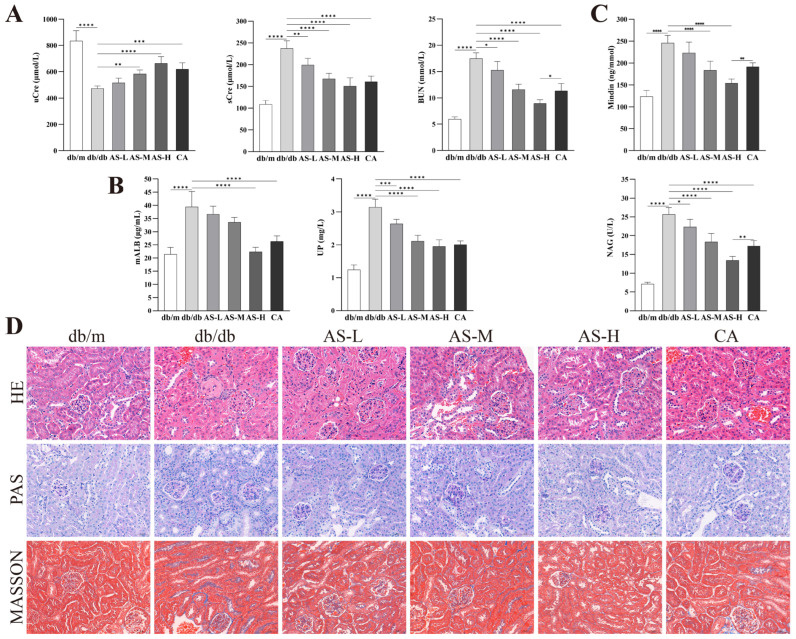
Effects of AS on renal function, injury markers, and renal pathology in db/db mice. (**A**,**B**) Renal function parameters: uCre, sCre, BUN, mALB, UP. (**C**) Podocyte injury marker Mindin and tubular injury marker NAG. (**D**) Representative images of renal tissue sections stained with HE, PAS, and MASSON trichrome (Scale bar = 50 μm). All data are presented as mean ± SD, *n* = 6 mice per group. Intergroup comparisons: * *p* < 0.05, ** *p* < 0.01, *** *p* < 0.001, **** *p* < 0.0001.

Analysis of serum and urine samples from different experimental groups revealed that AS intervention significantly increased uCre excretion, while decreasing sCre, BUN, mALB and UP levels in db/db mice (Figure 1A,B). These findings demonstrate the renoprotective efficacy of the AS combination. Furthermore, measurement of the podocyte injury marker Mindin and the tubular injury marker NAG in urine samples showed that AS significantly reduced their aberrantly high expression in the DKD state (Figure 1C), indicating that the renal protective effect of AS is closely associated with alleviation of podocyte and tubular damage. Notably, AS demonstrated superior efficacy compared to the conventional DKD medication canagliflozin (CA) in lowering BUN, Mindin, and NAG levels. Histopathological examination of kidney tissues (HE, PAS, and Masson staining) further corroborated that AS effectively ameliorated renal tubular damage, glomerular lesions, and collagen fiber deposition in DKD kidneys (Figure 1D).

### 2.2. Flavonoids Are the Primary Active Compounds in AS-Medicated Serum

**Figure 2:**
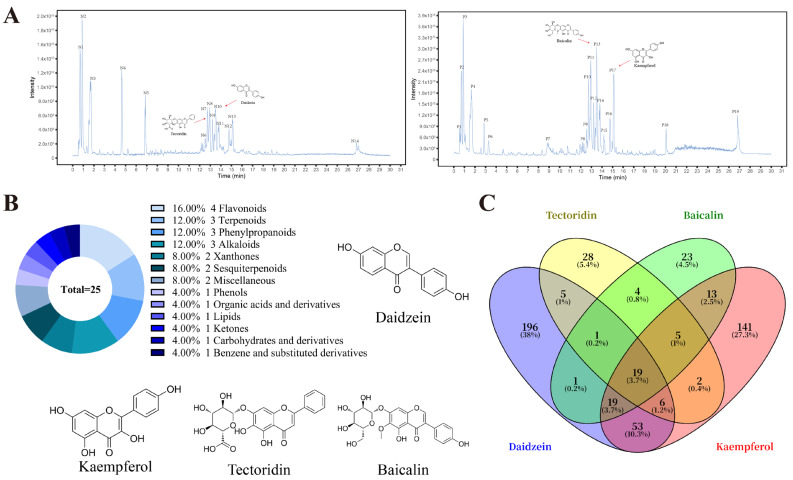
UHPLC-QE-MS analysis of active compounds in AS-medicated serum and their potential targets. (**A**) Total ion chromatograms (TIC) of AS-medicated serum in positive and negative ion modes (*n* = 3 mice per group, Supplementary Figure S1). (**B**) Class distribution analysis of major active compounds in AS-medicated serum. (**C**) Venn diagram illustrating potential targets of flavonoids derived from AS-medicated serum and their common targets.

Untargeted metabolomic analysis using ultra-high-performance liquid chromatography coupled with UHPLC-QE-MS was performed on serum samples obtained following AS intervention (AS-medicated serum). This analysis identified a total of 33 potential active compounds (Figure 2A, Supplementary Table S1). Among these, 25 major compounds exhibited a Composite Score > 0.8. Class analysis of these major compounds revealed that flavonoids constituted the predominant class, primarily comprising Daidzein, Kaempferol, Tectoridin, and Baicalin (Figure 2B, Table 1). By integrating data from the PubChem, ETCM, and TCMSP databases, 561 potential targets for these flavonoids were screened. Among these targets, 19 were identified as common targets (Figure 2C).

### 2.3. Potential Targets and Functional Insights of Flavonoids in AS for Treating DKD

**Figure 3:**
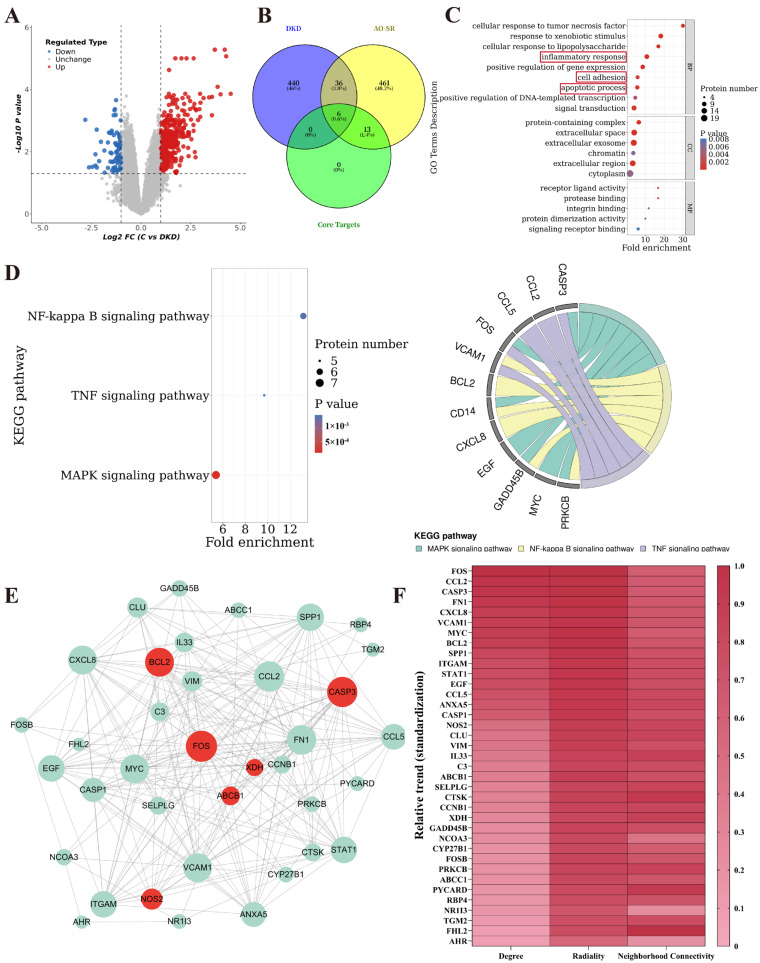
Analysis of DKD DEGs and potential mechanisms of AS action. (**A**) Volcano plot of DKD DEGs (Red: Upregulated genes; Green: Downregulated genes). (**B**) Venn diagram showing common differentially expressed genes (co-DEGs) associated with AS intervention in DKD and common core differentially expressed genes (co-core DEGs). (**C**) Bubble plot of GO enrichment analysis for co-DEGs (highlighting key biological processes). (**D**) KEGG pathway enrichment analysis for co-DEGs (Bar plot, bubble plot, and chord plot; core pathways post-filtering are indicated). (**E**) PPI network of co-DEGs (Node size proportional to Degree; Light green nodes: co-DEGs; Dark red nodes: co-core DEGs). (**F**) Heatmap based on PPI network topological parameters (Degree, Radiality, Neighborhood Connectivity) (Higher values indicate greater centrality).

Analysis of the GSE30529-GPL571 dataset (comprising 10 DKD samples and 12 control samples) identified 482 differentially expressed genes (DEGs) (360 upregulated, 122 downregulated) using thresholds of log2FC > 1 and adjusted *p*-value < 0.05. The DEGs are visualized in a volcano plot (Figure 3A). Venny 2.1 analysis revealed 42 common differentially expressed genes (co-DEGs) between the DKD DEGs and the potential targets of flavonoids in AS, which included 6 flavonoid co-core DEGs (Figure 3B). Gene Ontology (GO) and Kyoto Encyclopedia of Genes and Genomes (KEGG) pathway enrichment analyses of the 42 co-DEGs showed significant enrichment in biological processes highly relevant to DKD, such as inflammatory response, cell adhesion, and apoptosis (Figure 3C), as well as 44 KEGG pathways (Supplementary Figure S2). Further filtering (*p* < 0.01) identified the NF-κB signaling pathway, TNF signaling pathway, and MAPK signaling pathway as the central pathways regulated by these co-DEGs in DKD (Figure 3D). Detailed information on enriched GO/KEGG terms is provided in Supplementary Table S2. A protein–protein interaction (PPI) network illustrated the interactions among the 42 co-DEGs (Figure 3E). Based on topological parameters derived from PPI network analysis (Degree, Radiality, Neighborhood Connectivity), the study identified FOS, CASP3, and BCL2 among the 6 flavonoid co-core DEGs as hub nodes within the PPI network (Figure 3F, Supplementary Table S3).

### 2.4. Gene Set Enrichment Analysis (GSEA) of DEGs

**Figure 4:**
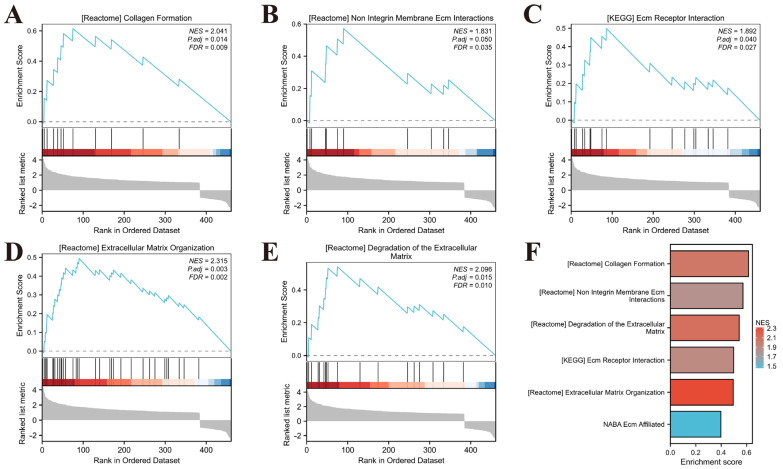
GSEA results for DEGs. (**A**–**E**) Representative GSEA enrichment plots for key pathways enriched in co-DEGs (Red indicates positive enrichment, while blue indicates negative enrichment.). (**F**) Bar plot of GSEA enrichment scores for representative pathways enriched in DEGs (Sorted by Enrichment Score; Bar color intensity is proportional to the NES).

The gene expression profiles from both DKD and control groups were imported into the GSEA analysis. Gene Ontology (GO) enrichment analysis was performed using the C2: Canonical Pathways (CP) gene set collection. Significantly enriched pathways were identified using screening thresholds of nominal *p*-value < 0.05 and FDR *q*-value < 0.25. The following pathways exhibited significant enrichment: Collagen Formation (Figure 4A, NES = 2.041, FDR = 0.009); Non Integrin Membrane ECM Interactions (Figure 4B, NES = 2.096, FDR = 0.010); ECM Receptor Interaction (Figure 4C, NES = 1.892, FDR = 0.027); Extracellular Matrix Organization (Figure 4D, NES = 2.315, FDR = 0.002); Degradation of the Extracellular Matrix (Figure 4E, NES = 2.096, FDR = 0.010).

### 2.5. Single-Cell RNA Sequencing Analysis of DKD Kidneys Reveals Spatial Distribution of Core Target Genes

**Figure 5:**
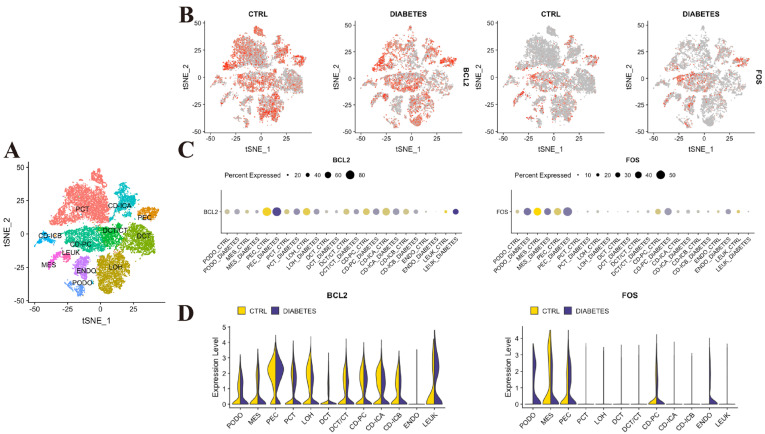
Single-cell RNA sequencing analysis of core target genes with diagnostic potential (BCL2, FOS) in DKD kidneys (Data source: KIT database). (**A**) UMAP plot illustrating cell type distribution in DKD patient kidneys (Data source: KIT database). (**B**) Relative expression levels of target genes (BCL2, FOS) across major kidney cell clusters (Color intensity indicates mean expression level). (**C**) Expression distribution of target genes across major kidney cell clusters (Dot plot: Dots represent individual cells). (**D**) Expression density distribution of target genes in podocytes versus tubular epithelial cells (Ridge plot: Peak height represents expression frequency).

In order to gain a deeper understanding of the spatial distribution of the 6 co-core-DEGs (CASP3, FOS, BCL2, NOS2, ABCB1 and XDH) in the kidneys of DKD, and to clarify the potential precise action sites of AS intervention, we conducted an analysis using the kidney single-cell RNA sequencing database KIT (Figure 5A). The key findings are as follows: BCL2 was widely expressed across kidney tissue in DKD patients; however, its expression levels were significantly reduced in podocytes and tubular epithelial cells compared to other cell types (Figure 5B,C). The expression distribution of CASP3, NOS2, ABCB1 and XDH within DKD kidney tissue showed no significant specificity (Supplementary Figure S3). Notably, FOS expression was not prominent in tubular epithelial cells of DKD kidneys but was specifically and highly elevated in podocytes (Figure 5D). These findings suggest that FOS may play a pivotal role in the pathogenesis of DKD by specifically regulating podocyte apoptosis.

### 2.6. Molecular Docking Simulation of Core Flavonoids in AS with FOS

**Figure 6:**
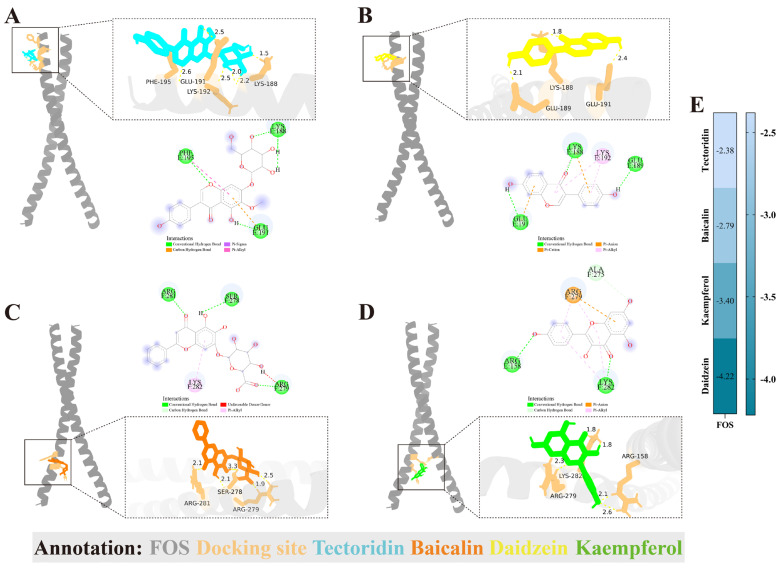
Molecular docking simulations of core flavonoids in AS with the FOS protein. (**A**) Three-dimensional (3D) and two-dimensional (2D) interaction diagrams of the FOS-Tectoridin docked complex (depicting binding site, interaction types, and distances). (**B**) 3D and 2D interaction diagrams of the FOS-Daidzein docked complex. (**C**) 3D and 2D interaction diagrams of the FOS-Baicalin docked complex. (**D**) 3D and 2D interaction diagrams of the FOS-Kaempferol docked complex (highlighting interactions of Kaempferol with both chains of the FOS dimer). (**E**) Minimum binding energy heat map of each compound and FOS.

The specific upregulation of FOS in diseased podocytes, as revealed by scRNA-seq (KIT Database), suggests its pivotal role in podocytopathy in DKD. Given that FOS functions as a transcription factor capable of modulating gene expression programs governing apoptosis, we hypothesized that the protective effects of AS might be mediated through the direct interaction of its bioactive flavonoids with FOS, thereby inhibiting its activity. To computationally assess the structural feasibility and binding affinity of this potential interaction, we performed molecular docking simulations (AutoDockTools-1.5.7). Figure 6A–D presents a schematic diagram illustrating the lowest binding energy of Tectoridin, Daidzein, Baicalin, and Kaempferol to FOB. The matrix heatmap of the interaction results was plotted using the binding energy (Figure 6E).

### 2.7. AS Significantly Ameliorates ECM Deposition and Apoptosis in Kidneys of db/db Mice

**Figure 7:**
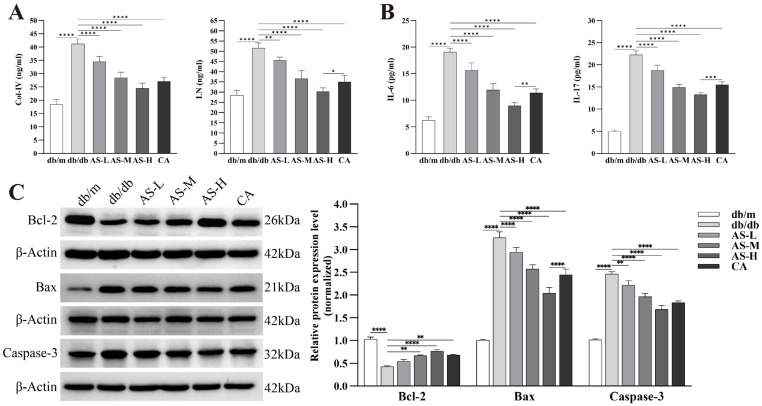
Effects of AS on ECM deposition and apoptosis-related molecules in kidneys of db/db mice. (**A**) Serum levels of key ECM components: Col-IV and LN. (**B**) Serum levels of pro-inflammatory cytokines: IL-6 and IL-17. (**C**) Expression of apoptosis-related proteins in kidney tissue: Anti-apoptotic protein Bcl-2, pro-apoptotic protein Bax, and cleaved Caspase-3 (detected by WB). All data are presented as mean ± SD, *n* = 3 mice per group. Intergroup comparisons: * *p* < 0.05, ** *p* < 0.01, *** *p* < 0.001, **** *p* < 0.0001.

Building upon previous findings, the therapeutic effect of AS on DKD is likely associated with the attenuation of podocyte apoptosis and the regulation of ECM homeostasis, potentially through the inhibition of the MAPK/FOS signaling pathway. To validate this hypothesis, we first measured the serum levels of ECM-related markers Col-IV, LN and pro-inflammatory cytokines IL-6, IL-17 in db/db mice. The results demonstrated that AS intervention significantly reduced the levels of Col-IV, LN, IL-6, and IL-17 in the serum of db/db mice (Figure 7A,B). Furthermore, Western blot (WB) analysis of kidney tissue samples from db/db mice revealed that, compared to the control group, the expression of pro-apoptotic proteins Bax and cleaved Caspase-3 was significantly increased, while the expression of the anti-apoptotic protein Bcl-2 was significantly decreased. Notably, AS intervention effectively reversed these dysregulations in apoptosis-related proteins in a dose-dependent manner (Figure 7C).

### 2.8. AS Regulates the MAPK/FOS Signaling Pathway in Kidneys of db/db Mice

**Figure 8:**
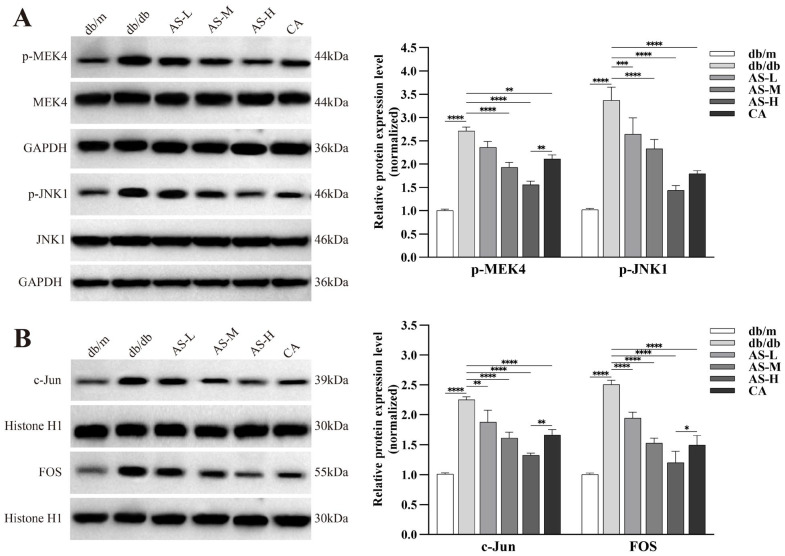
Effects of AS on key protein expression in the MAPK/FOS signaling pathway in kidneys of db/db mice. (**A**) Western blot analysis and quantification of upstream MAPK pathway activation markers: phosphorylated MEK4 (p-MEK4) and phosphorylated JNK1 (p-JNK1). (**B**) Western blot analysis and quantification of downstream MAPK pathway transcription factors: c-Jun and FOS. All data are presented as mean ± SD, *n* = 3 mice per group. Intergroup comparisons: * *p* < 0.05, ** *p* < 0.01, *** *p* < 0.001, **** *p* < 0.0001.

It is well-established that FOS, acting as a downstream effector in the MAPK cascade network, is primarily activated upon phosphorylation of MEK4 and JNK1. To further investigate the activation of the MAPK signaling pathway and its downstream transcriptional regulation, we examined the expression of key proteins. The findings revealed: Under DKD conditions, the protein expression levels of phosphorylated MEK4 (p-MEK4) and phosphorylated JNK1 (p-JNK1) were aberrantly elevated, indicating hyperactivation of this pathway. AS intervention dose-dependently and significantly reduced the protein expression levels of p-MEK4 and p-JNK1 in kidney tissues of db/db mice (Figure 8A). A similar pattern was observed for the protein expression of the key downstream transcription factors c-Jun and FOS: AS intervention also significantly and dose-dependently decreased the protein levels of c-Jun and FOS in the kidneys of db/db mice (Figure 8B).

## 3. Discussion

DKD is a common and serious complication of DM, posing a significant threat to patient health and quality of life [19]. Current management of DKD primarily relies on conventional medical approaches, including glycemic control, blood pressure regulation, and blockade of the renin–angiotensin–aldosterone system (RAAS) [20]. Although emerging therapeutics such as SGLT2 inhibitors and glucagon-like peptide-1 (GLP-1) receptor agonists are available, factors such as the potential risk of genitourinary tract infections with SGLT2 inhibitors, their restricted use in DKD patients with significantly reduced glomerular filtration rates, and gastrointestinal tolerability issues with GLP-1 receptor agonists that may hinder long-term adherence, collectively underscore the need for additional strategies to improve the prognosis and quality of life of DKD patients [21,22]. For the long-term management of chronic conditions like DM and DKD, promoting positive health behaviors to improve health outcomes is crucial [23]. Within this context, the use of dietary supplements represents one key compounds of active health behavior engagement for DKD patients [24]. Supplements containing vitamins, minerals, and Traditional Chinese herbs have been investigated for their potential benefits in various diseases [25]. Flavonoids are a critical subclass of polyphenols. A clinical meta-analysis on DKD, incorporating a qualitative synthesis of 17 independent studies, demonstrated that supplementation with natural dietary polyphenols and flavonoids primarily derived from Traditional Chinese herbs significantly improved renal function parameters in DKD patients [26]. Pathophysiological mechanisms in DKD, such as oxidative stress, inflammation, and dyslipidemia, provide a theoretical rationale for the application of flavonoid dietary supplements [27]. For instance, certain antioxidants have been shown to mitigate renal oxidative stress, while anti-inflammatory supplements may help alleviate the chronic low-grade inflammation associated with DKD [28,29]. However, most existing studies are limited in scope, often focusing on single supplements or relying on small-scale clinical trials. In this study, initial fundamental pharmacodynamic validation confirmed that the natural Traditional Chinese herbal dietary supplement combination AS ameliorates renal function and pathological damage in DKD animal models. In subsequent research, ultra-high-performance liquid chromatography coupled with UHPLC-QE-MS based untargeted metabolomics was first employed to identify the bioactive compounds within AS. This led to the screening of its four most prominent flavonoid compounds: Daidzein, Kaempferol, Tectoridin, and Baicalin. Multi-faceted bioinformatics analysis coupled with cross-validation revealed that the therapeutic mechanism of AS against DKD involves the targeted inhibition of the MAPK/FOS signaling pathway—associated with podocyte apoptosis—and its mediation of aberrant ECM accumulation. Experimental validation ultimately confirmed the validity of this mechanism.

Natural dietary supplements have garnered significant attention due to their potential multi-target bioactivities and relatively high safety profile [30]. Among these, flavonoids, a major class of polyphenolic widely present in fruits, vegetables, tea, legumes, and herbs, occupy a critically important position [31]. This prominence stems from their abundant dietary sources, diverse biological activities, and the accumulating positive evidence for their role in managing chronic diseases, particularly complications of metabolic disorders [32]. The outstanding value of flavonoids lies in their potent antioxidant and anti-inflammatory properties. These properties directly target core pathophysiological mechanisms underlying the development and progression of DKD [33]. A recent epidemiological study also confirmed that a long-term high intake of dietary flavonoids can effectively reduce the risk of DKD [34]. In the present study, we identified four major flavonoids in the AS combination: Daidzein, Kaempferol, Tectoridin, and Baicalin. Early clinical studies reported the renal function recovery effect of Daidzein in ESRD patients undergoing dialysis [35]. A study by Sandra et al. [36], involving a 7-month follow-up of 14 DKD patients, demonstrated that long-term soy protein intake led to Daidzein levels negatively correlating with urinary protein excretion rate. Liu et al. [37] conducted a 6-month randomized controlled trial (RCT) in 270 patients with declining renal function, ultimately confirming that daily Daidzein supplementation improved their renal function. Lu et al. [38] tracked 38,408 healthy women for 11.5 years and found that daily Kaempferol intake significantly reduced cancer risk; recent research by Okita et al. [39] indicated that enhancing mitochondrial metabolism and adenosine triphosphate (ATP) generation in human cells is a primary mechanism underlying Kaempferol’s protective effects. Baicalin, as a relatively well-studied flavonoid, has had its safety profile for long-term use and dose escalation confirmed by multiple RCTs [40,41]. Although clinical studies on Tectoridin are currently lacking, and existing studies on Daidzein, Kaempferol, and Baicalin also lack high-quality clinical evidence directly related to DKD, the growing focus on dietary therapy for DKD has prompted numerous researchers to initiate basic research. These studies aim to elucidate and evaluate Tectoridin’s impact on renal function [42] and the mechanisms of action of Daidzein [43], Kaempferol [44], and Baicalin [45] in DKD. This suggests that the AS herbal combination, as a natural dietary supplement, holds noteworthy potential value in the health management of DKD patients due to its multi-compounds and multi-effect characteristics. However, the clinical reports on these flavonoids are limited to small sample efficacy studies, and there is still a lack of high-quality clinical evidence. At the same time, in basic research, the application of animal and cell models is relatively monotonous, and multiple models need to be cross-validated. Therefore, it is still necessary to conduct in-depth and comprehensive research on flavonoid compounds.

The progressive development of DKD involves a complex network of cellular events, among which podocyte injury and loss constitute the central link in the disruption of the glomerular filtration barrier and the generation of proteinuria [46]. Recent research has profoundly revealed that podocyte apoptosis is not only a hallmark event in early DKD but also a key driver of the subsequent vicious cycle of glomerulosclerosis (characterized by ECM deposition) and chronic inflammation. As highly differentiated and terminally specialized cells, podocytes possess extremely limited regenerative capacity [47]. Their apoptotic loss leads to denudation of the GBM and abnormalities in the slit diaphragm structure, thereby compromising the integrity of the filtration barrier, significantly increasing proteinuria, and further exacerbating DKD progression [48]. Furthermore, apoptotic podocytes potently initiate and amplify local inflammatory responses [49]. By releasing pro-inflammatory cytokines (IL-6, IL-17), they foster a persistent inflammatory microenvironment within the kidney [50]. This microenvironment not only further promotes apoptosis of residual podocytes and other renal cells but also markedly stimulates the synthesis of collagen types I, III, IV, and fibronectin, resulting in excessive ECM accumulation, and ultimately accelerating the processes of glomerulosclerosis and tubulointerstitial fibrosis [51]. Within this intricate pathological cascade, the MAPK signaling pathway plays a pivotal role as a central hub regulating apoptosis, inflammation, and fibrosis [52]. Among its branches, the MEKK/JNK pathway serves as the primary route through which the MAPK cascade mediates classical apoptosis [53]. In DKD, phosphorylation-activated JNK stimulates the downstream transcription factor complex activator protein-1 (AP-1), upregulating the expression of the pro-apoptotic protein Bax while concurrently suppressing the function of the anti-apoptotic protein Bcl-2 [54]. This process directly initiates both mitochondrial-dependent and death receptor-mediated podocyte apoptotic programs [55].

**Figure 9:**
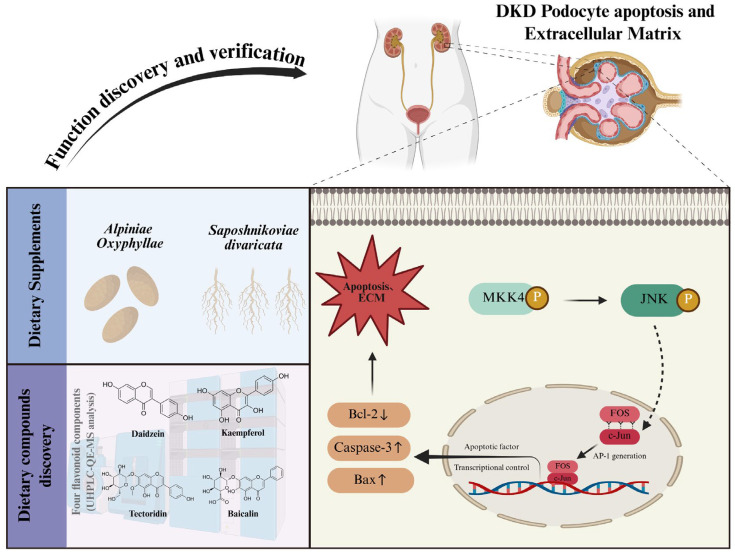
The dietary supplement AS is rich in flavonoid compounds and can effectively treat the podocyte apoptosis and ECM accumulation in DKD. The specific mechanism is related to the regulation of the MAPK/FOS signaling pathway (↑: Increase, ↓: Decrease).

Previous clinical research investigating podocyte apoptosis and the role of the MAPK signaling pathway in DKD has primarily focused on the initiation of the MAPK cascade (MEKK/JNK and its phosphorylation) and the detection of apoptotic phenotypes (expression of Bax, Bcl-2, Caspase-3) [56]. However, the decisive role of the transcription factor activator protein-1 (AP-1), a critical node within this complex regulatory network, in mediating podocyte apoptosis via the MAPK pathway in DKD remains to be fully elucidated [57]. As is well-established, the transcription factor AP-1 is a heterodimeric complex primarily composed of proteins from the c-Jun and FOS families [58]. Dysregulation of either c-Jun or FOS compounds impairs AP-1 complex formation, consequently diminishing its transcriptional activity [59]. Studies using in vitro models of high glucose-induced podocyte apoptosis have shown that increased apoptosis correlates with reduced expression of c-Jun and FOS [60]. This study, through bioinformatics analysis, identified the crucial role of the FOS protein in the apoptosis of podocytes in DKD. The FOS gene family comprises four members: FOS, FOSB, FOSL1, and FOSL2 [61]. Existing research indicates that targeted inhibition of FOSB attenuates apoptosis in podocytes and renal tubular epithelial cells (HK-2) induced by immunoglobulin A (IgA)-conditioned medium [62]. Furthermore, FOSB pro-apoptotic role has also been reported in an HK-2 cell apoptosis model induced by hypoxia-reoxygenation [63]. FOSL1 and FOSL2 have been shown to promote apoptosis in renal cells of mice with sepsis-associated nephritis [64] and in podocytes of mice with nephrotic syndrome [65]. Bioinformatic analysis of clinical data from chronic kidney disease (CKD) patients has suggested FOS as a core regulatory gene governing pan-apoptosis in the kidneys of CKD patients [66]. Additionally, the pro-apoptotic function of FOS has been confirmed in in vitro models of mesangial cell proliferative nephritis. Collectively [67], these studies demonstrate a close association between FOS and its family members and apoptosis in major intrinsic renal cells (mesangial cells, podocytes, renal tubular epithelial cells). Regrettably, despite the potential of FOS and its family members as key mediators of renal cell apoptosis, research specifically focused on their roles in DKD remains insufficient. In this study, we identified and confirmed the involvement of FOS in renal apoptosis in db/db mice. Further analyses, including ROC and single-cell RNA sequencing, revealed the clinical relevance of FOS in DKD and precisely localized its expression to podocytes in the kidneys of DKD patients. These findings provide critical and actionable leads for delving deeper into the precise regulatory mechanisms of the MAPK signaling pathway in podocyte apoptosis during DKD. Figure 9 visually demonstrates the regulatory role of the MAPK/FOS signaling pathway in podocyte apoptosis during DKD.

## 4. Materials and Methods

### 4.1. Animal Experimentation

#### 4.1.1. Reagents

There are two herbs of AS: *Alpiniae oxyphyllae*—derived from *Alpinia oxyphylla* Miq. (Zingiberaceae), whose medicinal part is Fructus Alpinae Oxyphyllae—and Saposhnikovia divaricata—derived from *Saposhnikovia divaricate* (Turcz.) Schischk. (Apiaceae), whose medicinal part is Radix Saposhnikoviae. Raw herbs of *Alpiniae oxyphyllae* and Saposhnikovia divaricata were purchased from Shenzhen Huahui Pharmaceutical Co., Ltd. (Shenzhen, China). AS extract was prepared in our laboratory according to the following procedure. After soaking *Alpiniae oxyphyllae* and Saposhnikovia divaricata in 10 times (*w*/*v*) of double-distilled water (ddH_2_O) for 30 min, the herbs were boiled for 1 h for extraction. The extracted solution was collected, and the herbs were then heated for an additional hour with 8 times (*w*/*v*) as much ddH_2_O. A final concentration of 1 g of raw herb per mL was achieved by combining, filtering, and concentrating the extracts that were obtained twice. The positive control drug, Canagliflozin (CA), was purchased from Xi’an Janssen Pharmaceutical Ltd. (Shanxi, China). Enzyme-linked immunosorbent assay (ELISA) kits for Type IV Collagen (Col-IV), Laminin (LN), Interleukin-6 (IL-6), Interleukin-17 (IL-17), Mindin, and NAG were obtained from Nanjing Jiancheng Bioengineering Institute (Nanjing, China. Catalogue Number: ml604125, ml063190, m1098430, m1037866, ml528541, ml037996).

#### 4.1.2. Experimental Animals and Intervention Protocol

Animals: Adult male db/db mice (6–8 weeks old) and their lean wild-type littermates (db/m) were purchased from GemPharmatech Co., Ltd. (Nanjing, Jiangsu, China) [Animal Production License No.: SCXK (Su) 2018-0008]. Animals were housed under specific pathogen-free (SPF) conditions in the Animal Experiment Center of Hainan Medical University [Facility Use License No.: SYXK (Qiong) 2022-0013]. Environmental parameters were maintained at: temperature 20–21 °C, relative humidity 46–58%, and a 12 h light/dark cycle. All animal procedures, including euthanasia, were performed in strict accordance with the guidelines approved by the Animal Welfare and Ethics Committee of Hainan Medical University (Approval No.: HYLL-2022-216, Approved on 20 April 2022), the Association for Assessment and Accreditation of Laboratory Animal Care International.

Model Confirmation and Grouping: db/db mice were confirmed as type 2 diabetes mellitus (T2DM) models (fasting blood glucose > 7.0 mmol/L on different days and random blood glucose ≥ 11.1 mmol/L [68]). Upon reaching 9 weeks of age, mice were randomly assigned to the following intervention groups (*n* = 6 per group): Model Control (db/db): Received vehicle (ultrapure water) only. AS Low-dose (AS-L): AS 1.3 g/kg/day. AS Medium-dose (AS-M): AS 2.6 g/kg/day. AS High-dose (AS-H): AS 5.2 g/kg/day. The adult dose of AS is 15 g/kg. Based on the dose conversion algorithm reported previously, the intragastric dose for mice was finally determined to be 2.6 g/kg/day [69]. Finally, the concentrations were adjusted by a factor of 2 for the low and high doses, respectively. Positive Control (CA): Canagliflozin 10 mg/kg/day [70]. Normal Control (db/m): Littermate db/m mice, received vehicle (ultrapure water). Intervention: All drugs/vehicle were dissolved in ultrapure water and administered once daily by oral gavage for 8 weeks.

Sample Collection and Processing: Urine Collection: Prior to the end of the intervention (before euthanasia), 24 h urine samples were collected from all mice. Euthanasia and Tissue Harvest: After 8 weeks of intervention, mice were deeply anesthetized with sodium pentobarbital. Whole blood was collected via femoral artery puncture. Serum was separated by centrifugation after clotting. Euthanasia was then confirmed by cervical dislocation. Both kidneys were rapidly excised. Sample Preservation: Urine and serum samples were stored at −20 °C until analysis. One kidney from each mouse was fixed in 4% paraformaldehyde (PFA) buffer for subsequent histological analysis. The contralateral kidney was snap-frozen in liquid nitrogen and stored at −80 °C for subsequent molecular biology studies. All the samples (kidneys, serum and urine) were used for pharmacological and pathological studies.

This study used a randomly generated sequence of computer-generated numbers to randomly assign animals to the experimental group and the control group. The randomization sequence was generated by researchers who were not involved in the subsequent intervention and result evaluation. During the experiment, blinding was implemented for the researchers conducting the animal experiments and data testing and statistical analysis, as they were unaware of the group allocation of the animals, in order to minimize measurement bias to the greatest extent. This study set clear humane endpoint criteria to minimize animal suffering. Animals would be euthanized once any of the following conditions occurred: (1) a weight loss of more than 20% from the peak; (2) severe behavioral abnormalities (such as lethargy, refusal to eat, loss of self-care ability); (3) breathing difficulties, ulcers or non-healing wounds; (4) non-fasting blood glucose persistently higher than 30 mmol/L accompanied by deterioration in health status; (5) severe edema or ascites.

#### 4.1.3. UHPLC-QE-MS Analysis

The samples (obtained from 3 mice randomly selected from each group) were centrifuged at 12,000 rpm for 15 min at 4 °C after being defrosted on ice for 30 s. After adding 1000 mL of extract (methanol/water = 4:1, IS = 1000:10) to 300 mL of supernatant in an EP tube, samples were sonicated in an ice water bath for 5 min, and then incubated at −40 °C for 1 h before being centrifuged at 12,000 rpm for 15 min at 4 °C. The supernatant was injected into the sample container and passed through a 0.22 μm filter membrane before being blended with 200 μL of each sample for machine detection. A Waters UPLC BEH C18 column (1.7 μm 2.1 × 100 mm) was used for the LC-MS/MS study on an Agilent ultra-high performance liquid chromatography 1290 UPLC system (Agilent Technologies, Santa Clara, CA, USA). A flow rate of 400 μL/min was applied, and a sample injection volume of 5 μL was used. Both 0.1% formic acid in water (A) and 0.1% formic acid in acetonitrile (B) consisted of the mobile phase. Elution follows a gradient linear procedure. Based on the IDA acquisition mode, the MS and MS/MS data were obtained using a Q Exactive Focus mass spectrometer and the Xcalibur software (version 4.2). The mass range for each acquisition cycle was 100 to 1500, and the top three results from each cycle were filtered before the matching MS/MS data were further obtained. Using the XCMS library search software (version 4.6.4), simple filtering of retention time and mass-to-charge ratio parameters was performed. Then, for different samples, peak alignment is carried out based on a retention time deviation of 0.5 min and a mass deviation of 10 ppm to ensure more accurate identification. Subsequently, peak extraction is conducted according to the set parameters such as mass deviation of 10 ppm, signal intensity deviation of 30%, signal-to-noise ratio of 3, peak width (5, 30), and summed ions, and the peak area is quantified. The target ions are then integrated, and the molecular formula is predicted through the molecular ion peak and fragment ions. This project includes QC samples and Blank samples. When quantifying each sample, the background of the Blank sample will be subtracted. After retention time correction, peaks with MS/MS data were identified. The compounds in AS extract were screened by combining the results of UHPLC-QE-MS analysis and HERB database (http://herb.ac.cn/, accessed on 20 July 2025). Finally, the substance name and its related information were determined through secondary mass spectrometry matching. The Composite Score [71] is adopted to enhance the overall credibility of the identification results. It mainly achieves this through weighted comprehensive scoring, integrating the evidence from three dimensions: the mass-to-charge ratio of metabolites, retention time, and the match degree of the secondary mass spectrum.

#### 4.1.4. Histopathological Staining

Hematoxylin and Eosin (HE) Staining: Deparaffinization was performed using xylene, followed by a series of ethanol washes until rehydration was achieved. The sections were stained with HE, rinsed with tap water, differentiated with hydrochloric acid-ethanol solution, and counterstained with eosin for 2 min. After a final rinse with running water, the samples were dehydrated in ethanol and cleared with xylene before being mounted with a neutral mounting medium.

Masson’s staining: Deparaffinization was performed using xylene, followed by a series of ethanol washes until rehydration was achieved. The sections were then incubated with potassium dichromate overnight, rinsed with running water, stained with Weigert’s iron hematoxylin, differentiated with 1% hydrochloric acid alcohol, and reblued. They were then stained with ponceau acid fuchsin, differentiated with phosphomolybdic acid, rinsed with 1% acetic acid, dehydrated in ethanol, and cleared with xylene before being mounted with neutral resin.

Periodic Acid-Schiff (PAS) Staining: Deparaffinized and rehydrated sections were oxidized in 0.5–1% periodic acid solution for 10 min, rinsed in distilled water, and then immersed in Schiff’s reagent (15–20 min). Sections were rinsed under tap water to develop the magenta color, briefly counterstained with Mayer’s hematoxylin, blued, dehydrated, cleared in xylene, and mounted. All sections were examined under the Leica DM4B upright digital research microscope (Leica, DMC6200, Wetzlar, Germany).

#### 4.1.5. Biochemical Indicator Analysis

Urinary creatinine (uCre), serum creatinine (sCre), blood urea nitrogen (BUN), urinary microalbumin (mALB), and urinary protein concentration (UP) were measured using an automated biochemistry analyzer (Beckman Instruments 1650, Brea, CA, USA). The total urinary protein excretion (UP, mg/24 h) was calculated using the following formula: UP (mg/24 h) = Urinary protein concentration (mg/mL) × 24 h urine volume (mL/24 h). Levels of the urinary podocyte injury marker Mindin, the urinary tubular injury marker NAG, as well as the serum ECM markers Col-IV and LN, and the pro-inflammatory cytokines IL-6 and IL-17, were determined using their respective ELISA kits (Serum samples were obtained from 3 mice randomly selected from each group.).

#### 4.1.6. Western Blot

Protein concentrations were determined using a BCA protein assay kit (P0012S, Beyotime, Shanghai, China). Equal amounts of protein were separated by SDS-PAGE and transferred onto a polyvinylidene difluoride (PVDF) membrane (Merck Millipore, Darmstadt, Germany). The membrane was then incubated overnight at 4 °C with primary antibodies against the following proteins: Bcl-2 (1:500; Proteintech, Rosemont, IL, USA), Bax (1:1000; Proteintech), cleaved Caspase-3 (1:1000; Cell Signaling Technology, Danvers, MA, USA), MEK4 (1:1000; Proteintech), p-MEK4 (1:1000; Proteintech), JNK1 (1:1000; Proteintech), p-JNK1 (1:1000; Proteintech), c-Jun (1:1000; Proteintech), Fos (1:1000; Proteintech), GAPDH (1:2000; Proteintech), Histone H1 (1:2000; Proteintech), and β-actin (1:10000; Pumei, Shanghai, China). After washing, the membrane was incubated with appropriate secondary antibodies at room temperature for 1 h. Protein bands were visualized using Clarity™ Western ECL substrate (1705061, Bio-Rad, Hercules, CA, USA) and quantified on a Bio-Rad ChemiDoc Touch imaging system (Bio-Rad). The integrated density value (IDV) of each band was measured after background subtraction. To normalize for loading variations, the IDV of the target protein was divided by the IDV of the corresponding housekeeping protein from the same sample [72]. Kidney tissue samples were obtained from 3 mice randomly selected from each group.

### 4.2. Bioinformatics Analysis

#### 4.2.1. Microarray Data Acquisition

The gene expression dataset GSE30529 [73], based on the Affymetrix HG-U133A 2.0 Array (GPL571), was retrieved from the Gene Expression Omnibus (GEO) database (https://www.ncbi.nlm.nih.gov/geo/, accessed on 15 July 2025). The search criteria included the keywords diabetic kidney disease/Homo sapiens/high-throughput gene expression profiling. The raw CEL files were processed and normalized in R (version 3.6.3) using the affy package. Specifically, we applied the RMA method for background adjustment, quantile normalization, and expression calculation. Probe-to-gene annotation was performed using the latest annotation file from the GPL571 platform [74]. For genes targeted by multiple probes, the expression values were averaged to create a unified gene-level expression measure. The normalized gene expression matrix was then log2-transformed and annotated using the hg38 reference genome to ensure the incorporation of the most up-to-date gene annotations for downstream analyses [75].

#### 4.2.2. Analysis of Differential Gene Expression

Differential expression analysis was conducted using the limma package in R (version 3.6.3). The significance of differential expression was assessed based on a moderated *t*-test. The resulting *p*-values were adjusted for multiple testing using the Benjamini–Hochberg method to control the false discovery rate (FDR). Genes with an adjusted *p*-value (adj. *p*) < 0.05 and an absolute log2 fold change (log2FC) > 1.0 were defined as differentially expressed genes (DEGs) [76]. Volcano plots were generated using the R packages ggplot2 (version 3.3.3) to visualize the identified DEGs.

#### 4.2.3. Screening of AS-Disease Common Targets

Potential targets of Daidzein, Kaempferol, Tectoridin, and Baicalin were retrieved and downloaded from the PubChem (https://pubchem.ncbi.nlm.nih.gov/, accessed on 20 July 2025), ETCM (http://www.tcmip.cn/ETCM/, accessed on 20 July 2025), and TCMSP (https://www.tcmsp-e.com/, accessed on 20 July 2025) databases using the respective compound names as keywords [77]. Screening was performed using the online tool Venny 2.1: AS-Disease Common Targets (co-DEGs): The intersection between the targets of each individual compound (Daidzein, Kaempferol, Tectoridin, or Baicalin) and the DKD DEGs was identified. AS-Disease Common Core Targets (co-core DEGs): The intersection between the common targets of all four compounds (the intersection of the targets of Daidzein, Kaempferol, Tectoridin, and Baicalin) and the DKD DEGs was identified.

#### 4.2.4. GSEA Enrichment Analysis and Single-Cell RNA Sequencing

GSEA was performed to evaluate the distribution patterns of gene sets and explore the biological phenotypes associated with differentially expressed genes (DEGs) in DKD. Functional enrichment analysis was conducted using the “clusterProfiler” package (version 3.14.3) in R and the C2 curated gene sets (c2.cp.v7.2.symbols.gmt) from the Molecular Signatures Database (MSigDB). Gene sets with an absolute normalized enrichment score (NES) > 1, a false discovery rate (FDR) *q*-value < 0.25, and a nominal *p*-value < 0.05 were considered significantly enriched. The online analysis database Kidney Interactive Transcriptomics (KIT, https://humphreyslab.com/SingleCell/, accessed on 20 July 2025) for single-cell data of kidneys provides validation analysis support for the finally selected co-core DEGs. The specific method is to compare the distribution and expression differences using the single-cell RNA sequencing data of healthy adult kidneys and diabetic patient kidneys in KIT. The Human Diabetic Kidney dataset was used in this study. In this dataset, 23,980 single-cell transcriptomes were generated from 3 control samples and 3 early DKD samples [78].

#### 4.2.5. GO and KEGG Pathway Enrichment Analysis of Genes

Gene Ontology (GO) and Kyoto Encyclopedia of Genes and Genomes (KEGG) pathway enrichment analyses were performed using the “clusterProfiler” package (version 3.14.3) in R, with the analysis species restricted to *Homo sapiens*. GO terms encompass three domains: Molecular Function (MF), Cellular Compounds (CC), and Biological Process (BP). The enrichment results were visualized using bubble plots and chord diagrams. Significantly enriched GO terms and KEGG pathways were selected based on an adjusted *p*-value (adj. *p*) < 0.05.

#### 4.2.6. PPI Network of Co-Core DEGs

The protein–protein interaction (PPI) network for all co-DEGs was constructed using the STRING online database (https://string-db.org/, accessed on 20 July 2025), with a filtering threshold of combined score > 0.4 [79]. Interaction data were subsequently downloaded, and the PPI network was optimized for visualization using Cytoscape software (version 3.9.1).

### 4.3. Statistical Analysis

All data are presented as mean ± standard deviation (SD) from at least three independent experiments. The sample size (*n*) per group of mice is indicated in the figure legends. Statistical significance between groups was determined using one-way analysis of variance (ANOVA), followed by Tukey’s post hoc test for multiple comparisons. The assumptions of normality (assessed by Shapiro–Wilk or Kolmogorov–Smirnov test) and homogeneity of variances (assessed by Brown-Forsythe or Bartlett’s test) were verified before performing ANOVA with *p* < 0.05 considered statistically significant. All statistical analyses were performed using GraphPad Prism software version 9.0 (San Diego, CA, USA).

## 5. Conclusions

Flavonoids, as the primary active components in AS, are likely the key mediators behind its significant alleviation of renal injury in db/db mice. This study highlights FOS, a core component of the AP-1 transcription factor complex known to regulate apoptosis and stress responses, as a promising and previously underexplored target in podocyte injury. Mechanistically, AS likely exerts its protective effects by modulating the MAPK/FOS signaling pathway, thereby mitigating podocyte apoptosis and ECM accumulation. These findings not only propose a novel regulatory role for FOS in DKD but also support the potential of AS, a flavonoid-rich natural product, as a dietary supplement strategy for DKD management.

However, this study still has certain limitations. For instance, the research has not yet confirmed the specific regulatory pathway of FOS in DKD by knocking out, overexpressing FOS, or directly blocking MAPK-mediated podocyte apoptosis. Additionally, the study mainly used db/db mice, which although constitute a stable DKD animal model, further cross-validation with multiple in vivo models and cell models is needed in subsequent research. This work provides the necessary experimental basis for future exploration of the specific role of FOS in podocyte apoptosis in DKD and the intervention measures based on AS.

## Figures and Tables

**Table 1:** Major flavonoids identified in AS-medicated serum.

Model	Name	Composite Score	Formula	Mzmed	Rtmed
NEG	Daidzein	0.9738	C_15_H_10_O_4_	253.0503	451.447
NEG	Tectoridin	0.9367	C_22_H_22_O_11_	461.1088	458.849
POS	Baicalin	0.9428	C_21_H_18_O_11_	447.0934	375.644
POS	Kaempferol	0.9174	C_15_H_10_O_6_	463.093	406.72

## Data Availability

The original contributions presented in this study are included in the article/Supplementary Materials. Further inquiries can be directed to the corresponding authors.

## Supplementary Materials

The following supporting information can be downloaded at: https://www.mdpi.com/article/10.3390/ph18101449/s1, Supplementary Table S1: Identification of Main Components of AS; Supplementary Table S2: co-DEGs GO and KEGG analysis of the original data; Supplementary Table S3: Co-DEGs PPI Network Node Raw Data; Supplementary Figure S1. TIC of AS-medicated serum in positive and negative ion modes; Supplementary Figure S2. The 44 KEGG classification diagrams enriched by co-DEGs; Supplementary Figure S3. Single-cell RNA sequencing analysis of core target genes with CASP3/ABCB1/NOS2/XDH in DKD kidneys.

## Abbreviations

The following abbreviations are used in this manuscript:

AS	*Alpiniae oxyphyllae*-*Saposhnikovia divaricata*
DM	Diabetes mellitus
DKD	Diabetic kidney disease
ESRD	End-stage renal disease
ECM	Extracellular matrix
DEGs	Differentially expressed genes
MAPK	Mitogen-activated protein kinase
CA	Canagliflozin
ELISA	Enzyme-linked immunosorbent assay
Col-IV	Type IV Collagen
LN	Laminin
IL-6	Interleukin-6
IL-17	Interleukin-17

## References

[1] Jia W., Chan J.C., Wong T.Y., Fisher E.B. (2025). Diabetes in China: Epidemiology, pathophysiology and multi-omics. Nat. Metab..

[2] American Diabetes Association Professional Practice Committee (2025). Diagnosis and Classification of Diabetes: Standards of Care in Diabetes—2025. Diabetes Care.

[3] Rayego-Mateos S., Rodrigues-Diez R.R., Fernandez-Fernandez B., Mora-Fernández C., Marchant V., Donate-Correa J., Navarro-González J.F., Ortiz A., Ruiz-Ortega M. (2022). Targeting inflammation to treat diabetic kidney disease: The road to 2030. Kidney Int..

[4] Młynarska E., Buławska D., Czarnik W., Hajdys J., Majchrowicz G., Prusinowski F., Stabrawa M., Rysz J., Franczyk B. (2024). Novel Insights into Diabetic Kidney Disease. Int. J. Mol. Sci..

[5] Feng L., Chen C., Xiong X., Wang X., Li X., Kuang Q., Wei X., Gao L., Niu X., Li Q. (2024). PS-MPs promotes the progression of inflammation and fibrosis in diabetic nephropathy through NLRP3/Caspase-1 and TGF-β1/Smad2/3 signaling pathways. Ecotoxicol. Environ. Saf..

[6] Vart P., Butt J.H., Jongs N., Schechter M., Chertow G.M., Wheeler D.C., Pecoits-Filho R., Langkilde A.M., Correa-Rotter R., Rossing P. (2024). Efficacy and Safety of Dapagliflozin in Patients With Chronic Kidney Disease Across the Spectrum of Frailty. J. Gerontol. Ser. A.

[7] Shulman R., Yang W., Cohen D.L., Reese P.P., Cohen J.B., Cohen D., Appel L.J., Chen J., Feldman H.I., Go A.S. (2024). Cardiovascular and Kidney Outcomes of Non-Diabetic CKD by Albuminuria Severity: Findings From the CRIC Study. Am. J. Kidney Dis..

[8] American Diabetes Association Professional Practice (2025). 1. Improving Care and Promoting Health in Populations: Standards of Care in Diabetes—2025. Diabetes Care.

[9] Jiang Y., Li Z., Yue R., Liu G., Yang M., Long C., Yan D. (2024). Evidential support for garlic supplements against diabetic kidney disease: A preclinical meta-analysis and systematic review. Food Funct..

[10] Kuszak A.J., Hopp D.C., Williamson J.S., Betz J.M., Sorkin B.C. (2016). Approaches by the US National Institutes of Health to support rigorous scientific research on dietary supplements and natural products. Drug Test. Anal..

[11] Rao Z., Zhou H., Li Q., Zeng N., Wang Q. (2024). Extraction, purification, structural characteristics and biological properties of the polysaccharides from Radix Saposhnikoviae: A review. J. Ethnopharmacol..

[12] Xiao M., Chen B., Niu K., Long Z., Yang F., Xie Y. (2022). Alpiniae oxyphylla fructus extract promotes longevity and stress resistance of C. elegans via DAF-16 and SKN-1. Front. Pharmacol..

[13] Yan Z.-J., Zhang L., Han X.-Y., Ma T.-P., Zhou S.-J. (2025). *Alpiniae oxyphyllae* Fructus-Saposhnikoviae Radix regulates NLRP3 inflammasome to ameliorate inflammatory response in diabetic kidney disease mice through PI3K/Akt/mTOR signaling pathway. China J. Chin. Mater. Medica.

[14] Wang X., Liu C., Jiang H., Chen B.-C., Yang X., Xiao M., Xie Y.-Q., Li K. (2023). Network pharmacology and verification experiment-based prediction of active components and potential targets of *Alpiniae oxyphyllae* Fructus-Saposhnikoviae Radix (Yizhiren-Fangfeng) for treatment of diabetic kidney disease. Tradit. Med. Res..

[15] Wang X., Jiang H., Wang F., Chang H., Rui K., Ma T., Zhao J., Liu C., Yang X., Xiao M. (2022). Based on the Method of Tonifying Kidney and Dispelling Wind Pathogen, the Effects of Alpinia Oxyphylla and Saposhnikoviae Radix and its Dismemberment Study on the Survival Rate and Inflammatory Factors of HK-2 Cells Induced by High Glucose were Studied. Lishizhen Med. Mater. Medica Res..

[16] Xie Y., Xiao M., Ni Y., Jiang S., Feng G., Sang S., Du G. (2018). *Alpinia oxyphylla* Miq. Extract Prevents Diabetes in Mice by Modulating Gut Microbiota. J. Diabetes Res..

[17] Kim C.W., Sung J.H., Kwon J.E., Ryu H.Y., Song K.S., Lee J.K., Lee S.R., Kang S.C. (2019). Toxicological Evaluation of Saposhnikoviae Radix Water Extract and its Antihyperuricemic Potential. Toxicol. Res..

[18] Gong Y.X. (2025). Research progress of effective components of traditional Chinese medicine in intervening apoptosis of renal tubular epithelial cells in diabetic kidney disease. J. Ethnopharmacol..

[19] Gaddy A., Elrggal M., Madariaga H., Kelly A., Lerma E., Colbert G.B. (2025). Diabetic Kidney Disease. Dis. Mon..

[20] Bae J.H. (2025). SGLT2 Inhibitors and GLP-1 Receptor Agonists in Diabetic Kidney Disease: Evolving Evidence and Clinical Application. Diabetes Metab. J..

[21] Neuen B.L., Heerspink H.J., Vart P., Claggett B.L., Fletcher R.A., Arnott C., Costa J.d.O., Falster M.O., Pearson S.-A., Mahaffey K.W. (2024). Estimated Lifetime Cardiovascular, Kidney, and Mortality Benefits of Combination Treatment With SGLT2 Inhibitors, GLP-1 Receptor Agonists, and Nonsteroidal MRA Compared With Conventional Care in Patients With Type 2 Diabetes and Albuminuria. Circulation.

[22] Yen F.-S., Hwu C.-M., Liu J.-S., Wu Y.-L., Chong K., Hsu C.-C. (2024). Sodium–Glucose Cotransporter-2 Inhibitors and the Risk for Dialysis and Cardiovascular Disease in Patients With Stage 5 Chronic Kidney Disease. Ann. Intern. Med..

[23] American Diabetes Association Professional Practice (2024). 1. Improving Care and Promoting Health in Populations: Standards of Care in Diabetes—2024. Diabetes Care.

[24] Jiménez-Osorio A.S., García-Niño W.R., González-Reyes S., Álvarez-Mejía A.E., Guerra-León S., Salazar-Segovia J., Falcón I., de Oca-Solano H.M., Madero M., Pedraza-Chaverri J. (2016). The Effect of Dietary Supplementation With Curcumin on Redox Status and Nrf2 Activation in Patients With Nondiabetic or Diabetic Proteinuric Chronic Kidney Disease: A Pilot Study. J. Ren. Nutr..

[25] Liu Y., Fang M., Tu X., Mo X., Zhang L., Yang B., Wang F., Kim Y.-B., Huang C., Chen L. (2024). Dietary Polyphenols as Anti-Aging Agents: Targeting the Hallmarks of Aging. Nutrients.

[26] Macena M.d.L., Nunes L.F.d.S., da Silva A.F., Pureza I.R.O.M., Praxedes D.R.S., Santos J.C.d.F., Bueno N.B. (2022). Effects of dietary polyphenols in the glycemic, renal, inflammatory, and oxidative stress biomarkers in diabetic nephropathy: A systematic review with meta-analysis of randomized controlled trials. Nutr. Rev..

[27] Ma Y., Wang J., Fan J., Jia H., Li J. (2024). Interrelation of Natural Polyphenol and Fibrosis in Diabetic Nephropathy. Molecules.

[28] Yanowsky-Escatell F.G., Andrade-Sierra J., Pazarín-Villaseñor L., Santana-Arciniega C., De Jesus Torres-Vázquez E., Chávez-Iñiguez J.S., Zambrano-Velarde M.Á., Preciado-Figueroa F.M. (2020). The Role of Dietary Antioxidants on Oxidative Stress in Diabetic Nephropathy. Iran. J. Kidney Dis..

[29] Wang X., Liang Q.-F., Zeng X., Huang G.-X., Xin G.-Z., Xu Y.-H., Wang S.-M., Tang D. (2021). Effects of soy isoflavone supplementation on patients with diabetic nephropathy: A systematic review and meta-analysis of randomized controlled trials. Food Funct..

[30] Gurley B.J., Chittiboyina A.G., ElSohly M.A., Yates C.R., Avula B., Walker L.A., Khan S.I., Khan I.A. (2024). The National Center for Natural Products Research (NCNPR) at 30: A Legacy of Pioneering Research in Natural Products and Dietary Supplements. J. Diet. Suppl..

[31] Solnier J., Chang C., Pizzorno J. (2023). Consideration for Flavonoid-Containing Dietary Supplements to Tackle Deficiency and Optimize Health. Int. J. Mol. Sci..

[32] Dini I., Grumetto L. (2022). Recent Advances in Natural Polyphenol Research. Molecules.

[33] Liu P.-Y., Hong K.-F., Liu Y.-D., Sun Z.-Y., Zhao T.-T., Li X.-L., Lao C.-C., Tan S.-F., Zhang H.-Y., Zhao Y.-H. (2024). Total flavonoids of Astragalus protects glomerular filtration barrier in diabetic kidney disease. Chin. Med..

[34] Liu F., Nie J., Deng M.-G., Yang H., Feng Q., Yang Y., Li X., Li X., Yang X., Li W. (2023). Dietary flavonoid intake is associated with a lower risk of diabetic nephropathy in US adults: Data from NHANES 2007–2008, 2009–2010, and 2017–2018. Food Funct..

[35] Fanti P., Sawaya B.P., Custer L.J., Franke A.A. (1999). Serum levels and metabolic clearance of the isoflavones genistein and daidzein in hemodialysis patients. J. Am. Soc. Nephrol..

[36] Teixeira S.R., Tappenden K.A., Carson L., Erdman J.W., Jones R., Prabhudesai M., Marshall W.P. (2004). Isolated soy protein consumption reduces urinary albumin excretion and improves the serum lipid profile in men with type 2 diabetes mellitus and nephropathy. J. Nutr..

[37] Liu Z.-M., Ho S.C., Chen Y.-M., Tang N., Woo J. (2014). Effect of whole soy and purified isoflavone daidzein on renal function—A 6-month randomized controlled trial in equol-producing postmenopausal women with prehypertension. Clin. Biochem..

[38] Wang L., Lee I.-M., Zhang S.M., Blumberg J.B., E Buring J., Sesso H.D. (2009). Dietary intake of selected flavonols, flavones, and flavonoid-rich foods and risk of cancer in middle-aged and older women. Am. J. Clin. Nutr..

[39] Okita K., Mizokami T., Yasuda O., Ikeda Y. (2025). Acute kaempferol ingestion lowers oxygen uptake during submaximal exercise and improves high-intensity exercise capacity in well-trained male athletes. Physiol. Rep..

[40] Li M., Shi A., Pang H., Xue W., Li Y., Cao G., Yan B., Dong F., Li K., Xiao W. (2014). Safety, tolerability, and pharmacokinetics of a single ascending dose of baicalein chewable tablets in healthy subjects. J. Ethnopharmacol..

[41] Dong R., Li L., Gao H., Lou K., Luo H., Hao S., Yuan J., Liu Z. (2021). Safety, tolerability, pharmacokinetics, and food effect of baicalein tablets in healthy Chinese subjects: A single-center, randomized, double-blind, placebo-controlled, single-dose phase I study. J. Ethnopharmacol..

[42] Niu X., Song H., Xiao X., Yu J., Yu J., Yang Y., Huang Q., Zang L., Han T., Zhang D. (2022). Tectoridin alleviates lipopolysaccharide-induced inflammation via inhibiting TLR4-NF-kappaB/NLRP3 signaling in vivo and in vitro. Immunopharmacol. Immunotoxicol..

[43] Pan Y., Zhou M., Liu Z., Hao C., Zhai J., Liu R., Shi Z., Sun J., Wang X. (2024). Synthesis and activity of arylcoumarin derivatives with therapeutic effects on diabetic nephropathy. Arch. Pharm..

[44] Alshehri A.S. (2021). Kaempferol attenuates diabetic nephropathy in streptozotocin-induced diabetic rats by a hypoglycaemic effect and concomitant activation of the Nrf-2/Ho-1/antioxidants axis. Arch. Physiol. Biochem..

[45] Hu H., Li W., Hao Y., Peng Z., Zou Z., Liang W. (2024). Baicalin ameliorates renal fibrosis by upregulating CPT1α-mediated fatty acid oxidation in diabetic kidney disease. Phytomedicine.

[46] Zhong S., Wang N., Zhang C. (2024). Podocyte Death in Diabetic Kidney Disease: Potential Molecular Mechanisms and Therapeutic Targets. Int. J. Mol. Sci..

[47] Adeva-Andany M.M., Carneiro-Freire N. (2022). Biochemical composition of the glomerular extracellular matrix in patients with diabetic kidney disease. World J. Diabetes.

[48] Liu F., Yang Z., Li J., Wu T., Li X., Zhao L., Wang W., Yu W., Zhang G., Xu Y. (2024). Targeting programmed cell death in diabetic kidney disease: From molecular mechanisms to pharmacotherapy. Mol. Med..

[49] Gong L., Wang R., Wang X., Liu J., Han Z., Li Q., Jin Y., Liao H. (2023). Research progress of natural active compounds on improving podocyte function to reduce proteinuria in diabetic kidney disease. Ren. Fail..

[50] Yang C., Zhang Z., Liu J., Chen P., Li J., Shu H., Chu Y., Li L. (2023). Research progress on multiple cell death pathways of podocytes in diabetic kidney disease. Mol. Med..

[51] Liu S., Wang H., Yang B., Hou B., Sun L., Pang H., Wang H., Fan Y. (2023). CircTAOK1 regulates high glucose induced inflammation, oxidative stress, ECM accumulation, and apoptosis in diabetic nephropathy via targeting miR-142-3p/SOX6 axis. Environ. Toxicol..

[52] Tesch G.H., Ma F.Y., Nikolic-Paterson D.J. (2020). Targeting apoptosis signal-regulating kinase 1 in acute and chronic kidney disease. Anat. Rec..

[53] Ma L., Wu F., Shao Q., Chen G., Xu L., Lu F. (2021). Baicalin Alleviates Oxidative Stress and Inflammation in Diabetic Nephropathy via Nrf2 and MAPK Signaling Pathway. Drug Des. Dev. Ther..

[54] Gong X., Ivanov V.N., Hei T.K. (2016). 2,3,5,6-Tetramethylpyrazine (TMP) down-regulated arsenic-induced heme oxygenase-1 and ARS2 expression by inhibiting Nrf2, NF-kappaB, AP-1 and MAPK pathways in human proximal tubular cells. Arch. Toxicol..

[55] Zhong Y., Jin C., Han J., Zhu J., Liu Q., Sun D., Xia X., Zhang Y., Peng X. (2021). Diosgenin Protects Against Kidney Injury and Mitochondrial Apoptosis Induced by 3-MCPD Through the Regulation of ER Stress, Ca(2+) Homeostasis, and Bcl2 Expression. Mol. Nutr. Food Res..

[56] Li X.M., Ma G., Liu J.M., Zhang G.M., Ma K.M., Ding B.M., Liang W., Gao W.M. (2024). The regulatory effect and mechanism of traditional Chinese medicine on the renal inflammatory signal transduction pathways in diabetic kidney disease: A review. Medicine.

[57] Vervaeke A., Lamkanfi M. (2025). MAP Kinase Signaling at the Crossroads of Inflammasome Activation. Immunol. Rev..

[58] Li F., Tian J., Zhang L., He H., Song D. (2024). A multi-omics approach to reveal critical mechanisms of activator protein 1 (AP-1). Biomed. Pharmacother..

[59] Gazon H., Barbeau B., Mesnard J.-M., Peloponese J.-M. (2018). Hijacking of the AP-1 Signaling Pathway during Development of ATL. Front. Microbiol..

[60] Bai X., Geng J., Li X., Yang F., Tian J. (2014). VEGF-A inhibition ameliorates podocyte apoptosis via repression of activating protein 1 in diabetes. Am. J. Nephrol..

[61] Park H.J., Kim J.W., Cho B.-S., Chung J.-H. (2014). Association of FOS-like antigen 1 promoter polymorphism with podocyte foot process effacement in immunoglobulin a nephropathy patients. J. Clin. Lab. Anal..

[62] Liao Y., Wang Z., Wang L., Lin Y., Ye Z., Zeng X., Wei F. (2020). MicroRNA-27a-3p directly targets FosB to regulate cell proliferation, apoptosis, and inflammation responses in immunoglobulin a nephropathy. Biochem. Biophys. Res. Commun..

[63] Tang X., Shen C., Liu C., Gao J. (2025). Network expression analysis identifies and experimentally validates the involvement of Fosb in acute kidney injury. FASEB BioAdvances.

[64] Yao T., Zhang L., Fu Y., Yao L., Zhou C., Chen G. (2021). Saikosaponin-d Alleviates Renal Inflammation and Cell Apoptosis in a Mouse Model of Sepsis via TCF7/FOSL1/Matrix Metalloproteinase 9 Inhibition. Mol. Cell. Biol..

[65] Jin Y., Wang H., Xin Y., Zhang Y. (2025). Fbxo11 maintains mitochondrial function and prevents podocyte injury in adriamycin-induced nephropathy by mediating the ubiquitin degradation of Fosl2. Exp. Cell Res..

[66] Zhang W.-T., Ge H.-W., Wei Y., Gao J.-L., Tian F., Zhou E.-C. (2024). Molecular characterization of PANoptosis-related genes in chronic kidney disease. PLoS ONE.

[67] Guan T., Gao Q., Chen P., Fu L., Zhao H., Zou Z., Mei C. (2014). Effects of polycystin-1 N-terminal fragment fusion protein on the proliferation and apoptosis of rat mesangial cells. Mol. Med. Rep..

[68] Luo W., Tang S., Xiao X., Luo S., Yang Z., Huang W., Tang S. (2023). Translation Animal Models of Diabetic Kidney Disease: Biochemical and Histological Phenotypes, Advantages and Limitations. Diabetes Metab. Syndr. Obes. Targets Ther..

[69] Reagan-Shaw S., Nihal M., Ahmad N. (2008). Dose translation from animal to human studies revisited. FASEB J..

[70] Huang Q., Shi W., Wang M., Zhang L., Zhang Y., Hu Y., Pan S., Ling B., Zhu H., Xiao W. (2024). Canagliflozin attenuates post-resuscitation myocardial dysfunction in diabetic rats by inhibiting autophagy through the PI3K/Akt/mTOR pathway. iScience.

[71] Zhou Z., Luo M., Zhang H., Yin Y., Cai Y., Zhu Z.-J. (2022). Metabolite annotation from knowns to unknowns through knowledge-guided multi-layer metabolic networking. Nat. Commun..

[72] Lan J., Xu B., Shi X., Pan Q., Tao Q. (2022). WTAP-mediated N6-methyladenosine modification of NLRP3 mRNA in kidney injury of diabetic nephropathy. Cell. Mol. Biol. Lett..

[73] Chen Y., Liao L., Wang B., Wu Z. (2024). Identification and validation of immune and cuproptosis—Related genes for diabetic nephropathy by WGCNA and machine learning. Front. Immunol..

[74] Suo L., Dai W., Qin X., Li G., Zhang D., Cheng T., Yao T., Zhang C. (2022). Screening of primary open-angle glaucoma diagnostic markers based on immune-related genes and immune infiltration. BMC Genet..

[75] Pan B., Kusko R., Xiao W., Zheng Y., Liu Z., Xiao C., Sakkiah S., Guo W., Gong P., Zhang C. (2019). Similarities and differences between variants called with human reference genome HG19 or HG38. BMC Bioinform..

[76] Lecamwasam A., Mansell T., Ekinci E.I., Saffery R., Dwyer K.M. (2022). Blood Plasma Metabolites in Diabetes-Associated Chronic Kidney Disease: A Focus on Lipid Profiles and Cardiovascular Risk. Front. Nutr..

[77] Wu D., Lin Q., Wang Z., Huang H., Song X., Gao Y., Yang X., Wen K., Sun X. (2024). Mechanism of Xue-Jie-San treating Crohn’s disease complicated by atherosclerosis: Network pharmacology, molecular docking and experimental validation. Phytomedicine.

[78] Wilson P.C., Wu H., Kirita Y., Uchimura K., Ledru N., Rennke H.G., Welling P.A., Waikar S.S., Humphreys B.D. (2019). The single-cell transcriptomic landscape of early human diabetic nephropathy. Proc. Natl. Acad. Sci. USA.

[79] Pyne T., Ghosh P., Dhauria M., Ganguly K., Sengupta D., Nandagopal K., Sengupta M., Das M. (2022). Prioritization of human well-being spectrum related GWAS-SNVs using ENCODE-based web-tools predict interplay between PSMC3, ITIH4, and SERPINC1 genes in modulating well-being. J. Psychiatr. Res..

