# Supplementary material for: Integrating UHPLC-QE-MS and Bioinformatics with Experimental Validation Reveals MAPK/FOS-Mediated Podocyte Apoptosis as the Key Mechanism of Alpiniae oxyphyllae and Saposhnikovia divaricata in Treating Diabetic Kidney Disease
Source: Pharmaceuticals (Basel). 2025 Sep 27;18(10):1449. doi: 10.3390/ph18101449 (PMC12567254; doi:10.3390/ph18101449)
Supplement: Supplementary file 1 [file pharmaceuticals-18-01449-s001.zip › pharmaceuticals-3812592-supplementary.pdf]

Supplementary Material

1.Supplementary Figures and Tables

1.1 Supplementary Tables

Supplementary Table S1: Identification of Main Components of AS

Model	Name	CompositeScore	Formula	Class	mzmed	rtmed
NEG	Xylose	0.8946	$C_5H_{10}O_5$	Carbohydrates and derivatives	149.0442	39.0632
NEG	Catechol	0.8273	$C_6H_6O_2$	Phenols	109.0279	187.006
NEG	Gentisic acid	0.9081	$C_7H_6O_4$	Xanthones	153.018	198.544
NEG	2',4'-DIHYDROXYACETOPHENONE	0.8659	$C_8H_8O_3$	Benzene and substituted derivatives	151.0388	231.0815
NEG	Caffeic acid	0.6738	$C_9H_8O_4$	Phenylpropanoids	179.034	240.865
NEG	Glutamylphenylalanine	0.7421	$C_{14}H_{18}N_2O_5$	Carboxylic acids and derivatives	293.1244	246.427
NEG	Vanillin	0.6195	$C_8H_8O_3$	Phenols	151.0388	296.554
NEG	Ferulic Acid	0.9514	$C_{10}H_{10}O_4$	Phenylpropanoids	193.0498	354.101
NEG	Daidzein	0.9738	$C_{15}H_{10}O_4$	Flavonoids	253.0503	451.447
NEG	Tectoridin	0.9367	$C_{22}H_{22}O_{11}$	Flavonoids	461.1088	458.849
NEG	sweroside	0.8852	$C_{16}H_{22}O_9$	Terpenoids	357.1187	502.021
NEG	Genistein	0.7983	$C_{15}H_{10}O_5$	Flavonoids	269.0454	524.032
NEG	Atractylenolide III	0.6547	$C_{15}H_{20}O_3$	Sesquiterpenoids	247.1333	630.891
NEG	(2S,3R,5R,10R,13R,14S,17S)-2,3,14-trihydroxy-10,13-dimethyl-17-[(2R,3R,5R)-2,3,6-trihydroxy-5,6-dimethylheptan-2-yl]-2,3,4,5,9,11,12,15,16,17-decahydro-1H-cyclopenta[a]phenanthren-6-one	0.8195	$C_{28}H_{46}O_7$	Terpenoids	493.3132	867.788
POS	Indigo	0.8432	$C_{16}H_{10}O_2N_2$	Alkaloids	263.0819	442.966

Model	Name	CompositeScore	Formula	Class	mzmed	rtmed
POS	Demethylcoclaurine hydrochloride	0.9125	$C_{16}H_{17}NO_3$	Alkaloids	272.1286	318.937
POS	Austricine	0.8761	$C_{15}H_{18}O_4$	Alkaloids	263.1284	317.82
POS	Kaempferol	0.9174	$C_{15}H_{10}O_6$	Flavonoids	463.093	406.72
POS	Baicalin	0.9428	$C_{21}H_{18}O_{11}$	Flavonoids	447.0934	375.644
POS	khelloside	0.9543	$C_{19}H_{20}O_{10}$	Ketones	409.1118	360.511
POS	Methyl jasmonate	0.8017	$C_{13}H_{20}O_3$	Lipids	225.1489	440.1795
POS	[(1S,6S,7R)-6-acetyloxy-1-(3-methylbutanoyloxy)spiro[4a,5,6,7a-tetrahydro-1H-cyclopenta[c]pyran-7,2'-oxirane]-4-yl]methyl 3-methylbutanoate	0.9884	$C_{22}H_{32}O_8$	Miscellaneous	425.216	540.879
POS	(2R,3S,4S,5R,6S)-2-(hydroxymethyl)-6-[4-[(E)-3-hydroxyprop-1-enyl]phenoxy]oxane-3,4,5-triol	0.8329	$C_{15}H_{20}O_7$	Miscellaneous	330.1556	501.775
POS	N-Methylphenylalanine	0.9672	$C_{10}H_{13}NO_2$	Organic acids and derivatives	180.1022	159.434
POS	Xanthyletin	0.8995	$C_{14}H_{12}O_3$	Phenylpropanoids	246.1132	592.0595
POS	(10-acetyloxy-8,8-dimethyl-2-oxo-9,10-dihydropyrano[2,3-f]chromen-9-yl) (Z)-2-methylbut-2-enoate	0.9256	$C_{21}H_{22}O_7$	Phenylpropanoids	404.1747	569.949
POS	linderalactone	0.8613	$C_{15}H_{16}O_3$	Sesquiterpenoids	245.1175	447.163
POS	Atractylenolide I	0.9937	$C_{15}H_{18}O_2$	Sesquiterpenoids	231.1384	701.457
POS	Nardosinone	0.879	$C_{15}H_{22}O_3$	Terpenoids	251.1646	541.416
POS	Grosheimin	0.7892	$C_{15}H_{18}O_4$	Terpenoids	263.1281	414.171
POS	Androsterone	0.7437	$C_{19}H_{30}O_2$	Terpenoids	291.2321	992.518
POS	Artemisinin	0.691	$C_{15}H_{22}O_5$	Terpenoids	283.1528	490.41
POS	TAMOXIFEN10540-29-1	0.8762	$C_{26}H_{29}NO$	Xanthones	372.2392	243.907

Supplementary Table S2: co-DEGs GO and KEGG analysis of the original data

Category	Name	Count	PValue	Fold Enrichment	FDR
BP	signal transduction	11	2.96E-04	3.900138	0.021298
BP	response to xenobiotic stimulus	10	2.38E-09	18.18674	1.89E-06
BP	inflammatory response	10	2.23E-07	10.73523	5.88E-05
BP	positive regulation of gene expression	9	6.87E-06	8.535495	7.76E-04
BP	apoptotic process	8	3.95E-04	5.655633	0.026012
BP	positive regulation of transcription by RNA polymerase II	8	0.01205	3.078917	0.243108
BP	cellular response to tumor necrosis factor	7	9.70E-08	30.05864	3.84E-05
BP	cellular response to lipopolysaccharide	7	2.86E-06	16.90799	4.53E-04
BP	cell adhesion	7	0.001041	5.807394	0.051454
BP	positive regulation of DNA-templated transcription	7	0.003575	4.546685	0.100999
BP	cellular response to type II interferon	6	6.05E-07	35.67399	1.20E-04
BP	response to ethanol	6	3.98E-06	24.40852	5.25E-04
BP	negative regulation of transcription by RNA polymerase II	6	0.049384	2.904563	0.443891
BP	response to lipopolysaccharide	5	2.11E-04	16.56293	0.016659
BP	defense response to virus	5	0.001616	9.621616	0.070055
BP	regulation of apoptotic process	5	0.002043	9.022605	0.072182
BP	angiogenesis	5	0.002347	8.68468	0.074252
BP	positive regulation of apoptotic process	5	0.004895	7.069541	0.129065
BP	negative regulation of apoptotic process	5	0.022609	4.502543	0.312554
BP	immune response	5	0.025873	4.318081	0.346869
BP	chromatin remodeling	5	0.055299	3.380189	0.449008
CC	cytoplasm	21	0.005501	1.731762	0.111495
CC	cytosol	19	0.017377	1.646641	0.1626
CC	nucleus	19	0.041546	1.506608	0.272124
CC	extracellular exosome	17	2.33E-06	3.754248	3.05E-04
CC	plasma membrane	17	0.061453	1.503846	0.335429
CC	nucleoplasm	16	0.009027	1.928879	0.111495
CC	extracellular space	15	7.23E-06	3.977925	4.73E-04
CC	extracellular region	15	8.13E-05	3.210889	0.002663
CC	protein-containing complex	9	5.16E-05	6.448729	0.002255

Category	Name	Count	PValue	Fold Enrichment	FDR
CC	chromatin	8	0.006182	3.495986	0.111495
CC	cell surface	6	0.009362	4.487484	0.111495
CC	perinuclear region of cytoplasm	6	0.015426	3.960952	0.155445
CC	intracellular membrane-bounded organelle	6	0.049263	2.906765	0.293337
CC	endoplasmic reticulum	6	0.088516	2.449064	0.445982
CC	external side of plasma membrane	5	0.008697	5.994177	0.111495
MF	protein binding	38	9.55E-04	1.303451	0.098744
MF	identical protein binding	10	0.00937	2.642574	0.190521
MF	DNA-binding transcription factor activity	6	0.008268	4.618872	0.189546
MF	protein homodimerization activity	6	0.019156	3.741716	0.333857
MF	signaling receptor binding	5	0.008082	6.114393	0.189546
MF	calcium ion binding	5	0.066207	3.177168	0.673102
MF	protease binding	4	0.001619	16.77091	0.098744
MF	receptor ligand activity	4	0.001619	16.77091	0.098744
MF	integrin binding	4	0.004442	11.73963	0.189546
MF	protein dimerization activity	4	0.007045	9.938315	0.189546
MF	protein-containing complex binding	4	0.035352	5.397533	0.47921
MF	DNA-binding transcription activator activity, RNA polymerase II-specific	4	0.083765	3.779359	0.729955
MF	cysteine-type endopeptidase activator activity involved in apoptotic process	3	0.001036	61.25027	0.098744
MF	chemokine activity	3	0.005032	27.62267	0.189546
MF	cysteine-type endopeptidase activity	3	0.008545	21.02621	0.189546
MF	carboxylic acid transmembrane transporter activity	2	0.00622	313.0569	0.189546
MF	CCR5 chemokine receptor binding	2	0.014455	134.1672	0.271311
MF	proteoglycan binding	2	0.028706	67.08362	0.412021
MF	efflux transmembrane transporter activity	2	0.028706	67.08362	0.412021
MF	ABC-type xenobiotic transporter activity	2	0.028706	67.08362	0.412021
MF	RNA polymerase II core promoter sequence-specific DNA binding	2	0.044748	42.68958	0.574654

Category	Name	Count	PValue	Fold Enrichment	FDR
MF	ubiquitin-like protein ligase binding	2	0.050696	37.56683	0.589042
MF	xenobiotic transmembrane transporter activity	2	0.050696	37.56683	0.589042
MF	CCR chemokine receptor binding	2	0.058572	32.3852	0.642149
MF	protein phosphatase 2A binding	2	0.06053	31.30569	0.642149
MF	transcription coregulator binding	2	0.074132	25.38299	0.713784
MF	ATPase-coupled transmembrane transporter activity	2	0.076059	24.71502	0.713784
MF	chemoattractant activity	2	0.081817	22.9066	0.729955
MF	ABC-type transporter activity	2	0.087541	21.34479	0.73655
MF	transcription coactivator binding	2	0.097003	19.16675	0.788954
KEGG	Pathways in cancer	12	7.29E-06	5.19284	1.19E-04
KEGG	Lipid and atherosclerosis	10	1.88E-07	10.67818	6.10E-06
KEGG	Pertussis	9	8.31E-10	26.61331	1.08E-07
KEGG	Influenza A	9	4.52E-07	11.99906	1.18E-05
KEGG	AGE-RAGE signaling pathway in diabetic complications	8	1.66E-07	18.2692	6.10E-06
KEGG	Tuberculosis	8	8.85E-06	10.1384	1.28E-04
KEGG	Salmonella infection	8	7.04E-05	7.351351	5.80E-04
KEGG	Legionellosis	7	1.02E-07	28.83108	6.10E-06
KEGG	Amoebiasis	7	3.88E-06	15.67515	8.40E-05
KEGG	Toll-like receptor signaling pathway	7	5.39E-06	14.8123	1.00E-04
KEGG	Hepatitis B	7	5.37E-05	9.905157	5.37E-04
KEGG	NOD-like receptor signaling pathway	7	1.22E-04	8.542543	9.33E-04
KEGG	Chemical carcinogenesis - receptor activation	7	2.47E-04	7.509491	0.001772
KEGG	Coronavirus disease - COVID-19	7	4.26E-04	6.783784	0.002639
KEGG	Shigellosis	7	5.54E-04	6.458162	0.003273
KEGG	MAPK signaling pathway	7	7.13E-05	5.381802	0.00687
KEGG	MicroRNAs in cancer	7	1.75E-03	5.174809	0.007599
KEGG	Leishmaniasis	6	1.80E-05	17.51762	2.34E-04
KEGG	Colorectal cancer	6	2.88E-05	15.9068	3.41E-04
KEGG	Small cell lung cancer	6	3.98E-05	14.88056	4.31E-04
KEGG	Chagas disease	6	6.51E-05	13.43584	5.80E-04
KEGG	NF-kappa B signaling pathway	6	1.43E-03	13.17992	5.80E-04
KEGG	Yersinia infection	6	2.59E-04	10.0282	0.001772

**Category**	**Name**	**Count**	**PValue**	**Fold Enrichment**	**FDR**
KEGG	Herpes simplex virus 1 infection	6	9.20E-04	7.603802	0.004786
KEGG	Kaposi sarcoma-associated herpesvirus infection	6	0.001283	7.060673	0.006415
KEGG	Epstein-Barr virus infection	6	0.001533	6.783784	0.00687
KEGG	Human cytomegalovirus infection	6	0.002404	6.123415	0.009944
KEGG	Cytosolic DNA-sensing pathway	5	3.87E-04	13.8945	0.002513
KEGG	Rheumatoid arthritis	5	6.46E-04	12.1394	0.0035
KEGG	IL-17 signaling pathway	5	6.46E-04	12.1394	0.0035
KEGG	TNF signaling pathway	5	0.0015	9.69112	0.00687
KEGG	Apoptosis	5	0.002448	8.47973	0.009944
KEGG	Osteoclast differentiation	5	0.002936	8.064638	0.011567
KEGG	Breast cancer	5	0.003324	7.792184	0.012709
KEGG	Gastric cancer	5	0.003488	7.688288	0.012956
KEGG	Necroptosis	5	0.004297	7.253102	0.015096
KEGG	Neutrophil extracellular trap formation	5	0.008484	5.975354	0.028281
KEGG	Chemokine signaling pathway	5	0.008484	5.975354	0.028281
KEGG	Transcriptional misregulation in cancer	5	0.009266	5.824461	0.030116
KEGG	Focal adhesion	5	0.010095	5.681001	0.031245
KEGG	Pathogenic Escherichia coli infection	5	0.010095	5.681001	0.031245
KEGG	Human immunodeficiency virus 1 infection	5	0.011893	5.414288	0.034358
KEGG	Human papillomavirus infection	5	0.05027	3.463193	0.101393
KEGG	PI3K-Akt signaling pathway	5	0.064537	3.185755	0.119107

Supplementary Table S3: co-DEGs PPI Network Node Raw Data

name	Degree	Radiality	Neighborhood Connectivity
TGM2	3	0.680556	18
FHL2	2	0.701389	23.5
CYP27B1	5	0.743056	15.2
FOSB	5	0.763889	16.2
SELPLG	8	0.756944	19.75
GADD45B	6	0.770833	17.66667
IL33	10	0.777778	20.2
PYCARD	4	0.722222	21.5
RBP4	4	0.708333	16.75
C3	9	0.756944	18.11111
XDH	7	0.75	20.28571
NOS2	12	0.819444	19.08333
CLU	11	0.8125	19.45455
CTSK	8	0.791667	21.625
ITGAM	19	0.868056	17.89474
VIM	11	0.8125	17.45455
CASP1	16	0.847222	17.25
CXCL8	22	0.888889	16.27273
STAT1	19	0.875	17.84211
FOS	25	0.923611	14.64
CCL5	18	0.861111	18.5
VCAM1	22	0.888889	16.18182
CCNB1	8	0.770833	19.25
CCL2	24	0.902778	15.29167
NCOA3	6	0.743056	10.5

name	Degree	Radiality	Neighborhood Connectivity
AHR	2	0.513889	5
PRKCB	5	0.756944	19.6
MYC	22	0.902778	15.04545
ABCC1	5	0.743056	16.4
BCL2	22	0.895833	16.13636
SPP1	20	0.881944	16.85
NR1I3	4	0.631944	5.5
FN1	23	0.902778	15.73913
CASP3	24	0.909722	15.33333
ANXA5	17	0.861111	19
EGF	19	0.875	17.57895
ABCB1	9	0.8125	17.33333

1.2 Supplementary Figures

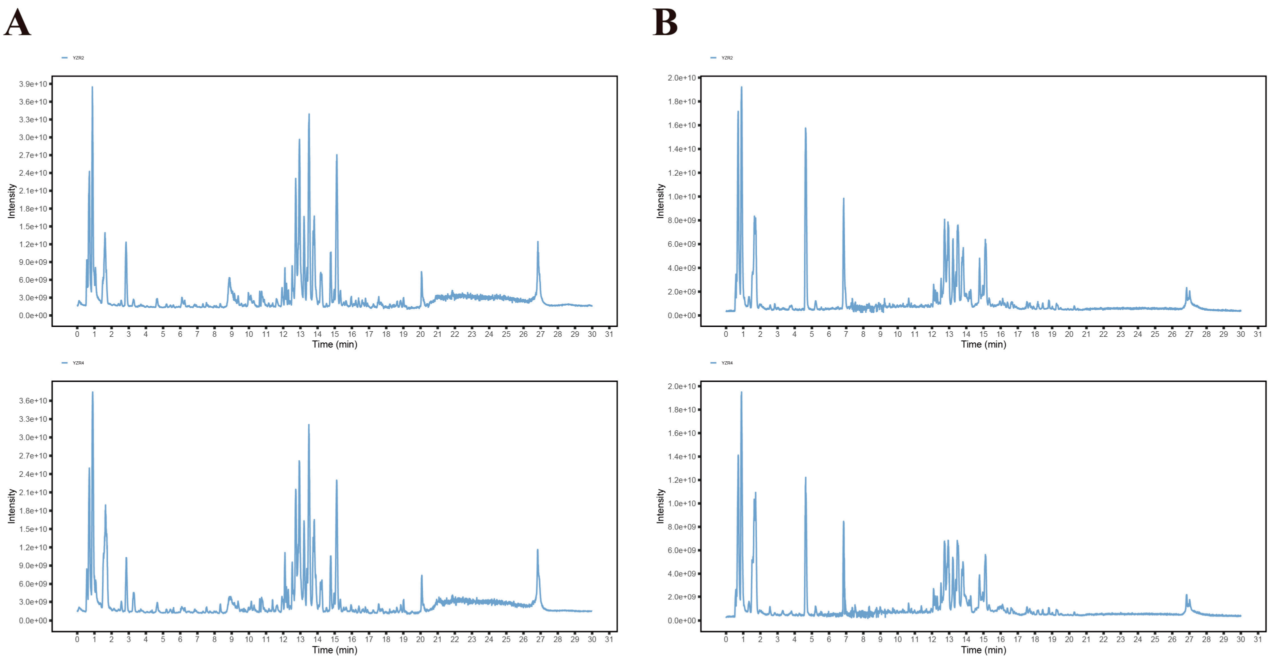

Supplementary Figure S1. Total ion chromatograms (TIC) of AS-medicated serum in positive and negative ion modes. (A) Positive ion mode. (B) Negative ion mode.

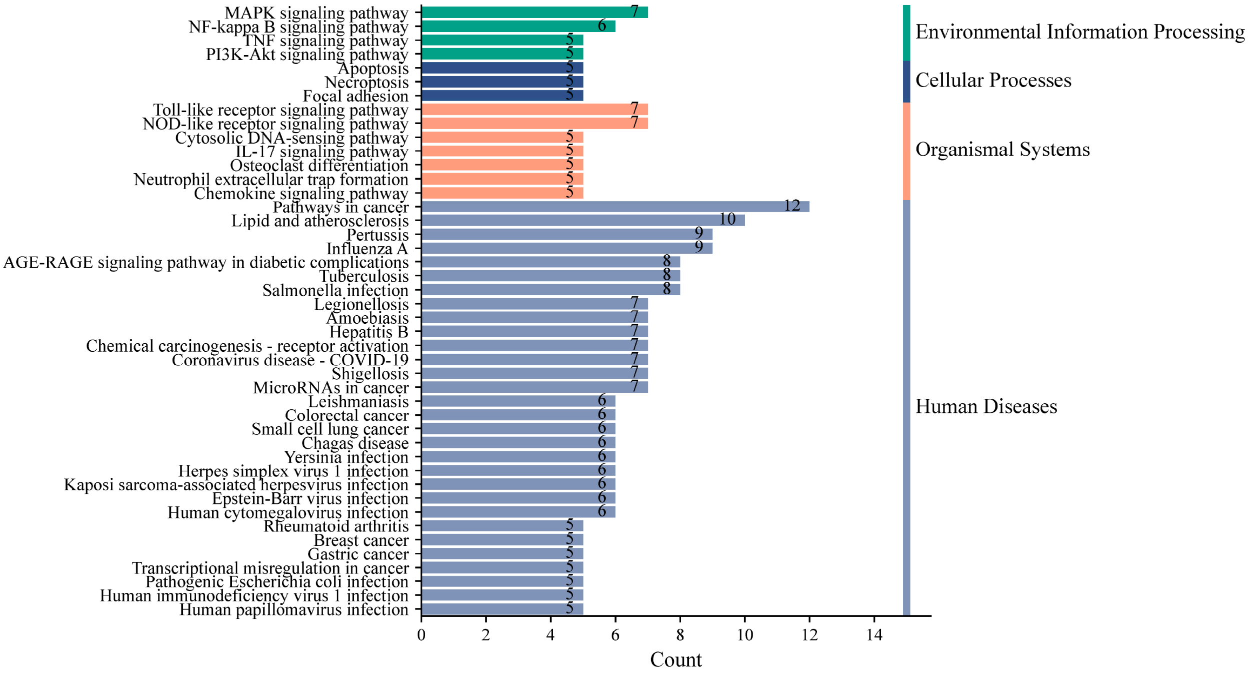

Supplementary Figure S2. The 44 KEGG classification diagrams enriched by co-DEGs.

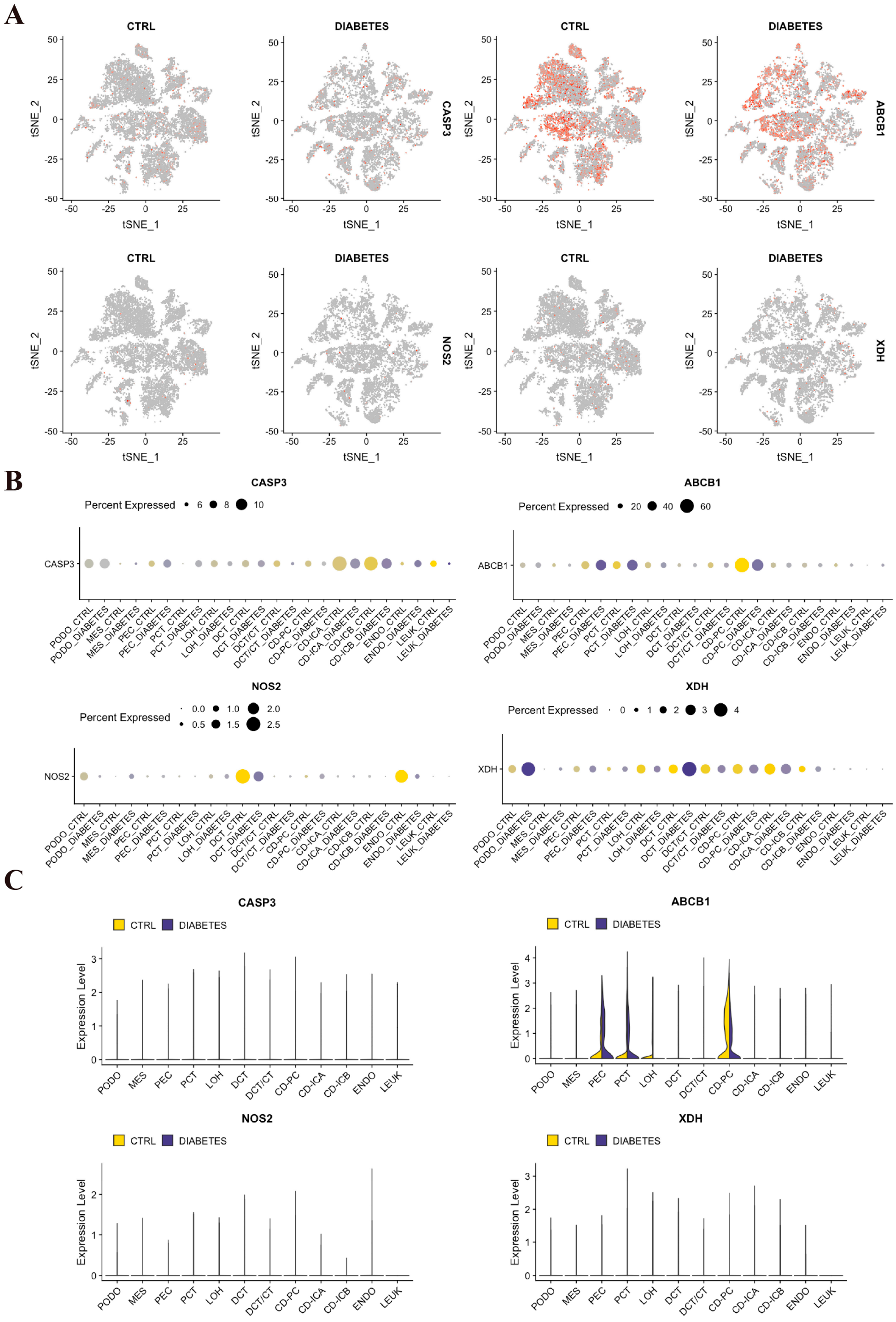

Supplementary Figure S3. Single-cell RNA sequencing analysis of core target genes with CASP3/ABCB1/NOS2/XDH in DKD kidneys (Data source: KIT database)**.** (A) Relative expression levels of CASP3/ABCB1/NOS2/XDH across major kidney cell clusters (Color intensity indicates mean expression level). (B) Expression distribution of target genes across major kidney cell clusters (Dot plot: Dots represent individual cells). (C) Expression density distribution of target genes in podocytes versus tubular epithelial cells (Ridge plot: Peak height represents expression frequency).
